# Astrovirus replication in human intestinal enteroids reveals multi-cellular tropism and an intricate host innate immune landscape

**DOI:** 10.1371/journal.ppat.1008057

**Published:** 2019-10-31

**Authors:** Abimbola O. Kolawole, Carmen Mirabelli, David R. Hill, Sophia A. Svoboda, Andrew B. Janowski, Karla D. Passalacqua, Benancio N. Rodriguez, Michael K. Dame, Pamela Freiden, Ryan P. Berger, Diem-lan Vu, Myra Hosmillo, Mary X. D. O’Riordan, Stacey Schultz-Cherry, Susana Guix, Jason R. Spence, David Wang, Christiane E. Wobus

**Affiliations:** 1 Department of Microbiology and Immunology, University of Michigan, Ann Arbor, Michigan, United States of America; 2 Division of Gastroenterology, Department of Internal Medicine, University of Michigan, Ann Arbor, Michigan, United States of America; 3 Department of Pediatrics, Washington University, St. Louis, Missouri, United States of America; 4 St. Jude Children’s Hospital, Memphis, Tennessee, United States of America; 5 Enteric Virus Laboratory, Department of Genetics, Microbiology and Statistics, University of Barcelona, Barcelona, Spain; 6 Division of Virology, Department of Pathology, University of Cambridge, Addenbrooke's Hospital, Cambridge, United Kingdom; 7 Department of Cell and Developmental Biology, University of Michigan, Ann Arbor, Michigan, United States of America; 8 Department of Biomedical Engineering, University of Michigan, Ann arbor, Michigan, United States of America; 9 Departments of Molecular Microbiology, and Pathology and Immunology, Washington University, St. Louis, Missouri, United States of America; University Heidelberg, GERMANY

## Abstract

Human astroviruses (HAstV) are understudied positive-strand RNA viruses that cause gastroenteritis mostly in children and the elderly. Three clades of astroviruses, classic, MLB-type and VA-type have been reported in humans. One limitation towards a better understanding of these viruses has been the lack of a physiologically relevant cell culture model that supports growth of all clades of HAstV. Herein, we demonstrate infection of HAstV strains belonging to all three clades in epithelium-only human intestinal enteroids (HIE) isolated from biopsy-derived intestinal crypts. A detailed investigation of infection of VA1, a member of the non-canonical HAstV-VA/HMO clade, showed robust replication in HIE derived from different patients and from different intestinal regions independent of the cellular differentiation status. Flow cytometry and immunofluorescence analysis revealed that VA1 infects several cell types, including intestinal progenitor cells and mature enterocytes, in HIE cultures. RNA profiling of VA1-infected HIE uncovered that the host response to infection is dominated by interferon (IFN)-mediated innate immune responses. A comparison of the antiviral host response in non-transformed HIE and transformed human colon carcinoma Caco-2 cells highlighted significant differences between these cells, including an increased magnitude of the response in HIE. Additional studies confirmed the sensitivity of VA1 to exogenous IFNs, and indicated that the endogenous IFN response of HIE to curtail the growth of strains from all three clades. Genotypic variation in the permissiveness of different HIE lines to HAstV could be overcome by pharmacologic inhibition of JAK/STAT signaling. Collectively, our data identify HIE as a universal infection model for HAstV and an improved model of the intestinal epithelium to investigate enteric virus-host interactions.

## Author summary

Human astroviruses (HAstV) are understudied positive-strand RNA viruses that typically cause gastroenteritis mostly in children and the elderly, but more recent studies also implicate them in neurological disease in immunocompromised patients. To better understand these viruses, a physiologically relevant cell culture model that supports growth of all clades of HAstV would be highly beneficial. Herein, we demonstrated robust infection of HAstV strains belonging to all three clades in epithelium-only human intestinal enteroids (HIE) isolated from biopsy-derived intestinal crypts from different patients and intestinal regions, making HIE a valuable model to study HAstV biology. Using this system, we identify for the first time that VA1 infects several cell types, including intestinal progenitor cells and mature enterocytes. Analysis of the antiviral host response to infection demonstrated that HIE respond to infection with a type I and III interferon response. This response reduced HAstV replication and when blocked resulted in increased infection. Establishment of the HIE system for HAstV research lays the foundation for future basic and translational discoveries.

## Introduction

Human astroviruses (HAstV) are a highly prevalent but understudied group of enteric viruses. AstV package a positive-sense RNA genome into non-enveloped, icosahedral capsids, and are transmitted by the fecal-oral route [1]. HAstV were first discovered in 1975 [1]. These viruses cause a range of symptoms from diarrhea to encephalitis, or asymptomatic infections and systemic, extra-intestinal infections are common [2]. The classical HAstV cause 2–9% of all acute nonbacterial gastroenteritis in children worldwide. Other high-risk groups include immunocompromised individuals and the elderly. Metagenomics surveillance studies continue to identify novel AstV in many animal species, highlighting their zoonotic potential. Currently, three groups of HAstV are recognized: classic HAstV (serotypes 1–8), and non-classic HAstV-MLB (Melbourne) (MLB1–3), and HAstV-VA/HMO (Virginia/Human-Mink-Ovine-like) (VA1–5). The clinical impact of the MLB and VA strains is poorly defined, and many fundamental features of their biology remain unknown or are incompletely understood, including the infected cell type(s) and the nature of the host immune response to infection. This has been in part due to the wide genetic diversity of these viruses and the unavailability for many years of cell culture systems for these non-classical HAstV. The VA1 strain can be successfully propagated in several continuous cell lines, including human embryonic kidney HEK293T, adenocarcinomic human lung epithelial A549, and human colon carcinoma Caco-2 cells, the most commonly used cell line to propagate all eight serotypes of classic HAstV, and primary astrocytes [3, 4]. Very recently, Vu et al. [5] reported the propagation of MLB1 and MLB2 strains in human liver Huh7 and A549, but not in Caco-2 cells. Although A549 cells may also be susceptible to infection by classic HAstV serotypes, they have not supported the replication of all tested serotypes [6]. Thus, a significant roadblock to a detailed understanding of these viruses is the lack of a robust, physiologically relevant viral propagation system for genetically diverse viruses from all clades.

Human intestinal organoids are a physiologically relevant model of the intestine that can be generated by expanding isolated patient-derived intestinal epithelial stem cells in 3-dimensional culture [7]. We will refer to those as human intestinal enteroids (HIE) throughout. In recent years, HIE are increasingly being used as disease models for the study of host-pathogen interactions, especially in the context of infection with viruses that lack a robust cell system such as rotaviruses and noroviruses [7–11]. HIE are grown in extracellular matrix scaffolds in 3D and maintain aspects of their *in vivo* physiology for long periods in culture [12]. The HIE system exhibits multiple advantages over traditional transformed intestinal epithelial cell lines (e.g., Caco-2 cells): 1. HIE only contain non-transformed human cells [10]; 2. they contain multiple cell types [13]; 3. they maintain the intestinal region-specific differences in culture [14]; 4. they can be differentiated into cell populations located in the villus or crypt regions [15]; and 5. HIE maintain the genetic identity of the host [10]. HIE proliferate when maintained in an undifferentiated crypt-like state in the presence of WNT3A [11]. These undifferentiated HIE express markers of cell types found in the crypt, such as stem cells (e.g., Leucine Rich Repeat Containing G Protein-Coupled Receptor 5 [LGR5]) and Paneth cells (e.g., Lysozyme) [15]. Withdrawal of WNT3A reduces proliferation and stem cell-like properties and induces differentiation into professional villus-like cell types that express markers of mature enterocytes (e.g., sucrase isomaltase) and goblet cells (e.g., mucin 2 [MUC2]) [15]. HIE have been successfully used to culture human rotaviruses and noroviruses that were refractive to growth in transformed cells through a method of breaking apart the 3D spheres or by separating and seeding cells into a 2D monolayer [7, 8]. Thus, we aimed to investigate HIE as a potentially universal HAstV culture model for the study of AstV infection parameters and antiviral host responses.

Our data demonstrate for the first time that HAstV strains belonging to all clades robustly infected HIE obtained from different intestinal segments (jejunum, duodenum, ileum, colon) and from donors of different age groups (fetal and adult). We further uncovered that VA1 infects multiple cell types in HIE, which include intestinal progenitor cells, representing the first time this cell type has been identified as a target cell for intestinal viruses. RNAseq analysis of VA1-infected HIE revealed that an interferon (IFN)-mediated innate antiviral response is the predominant host response to VA1 infection. Comparative studies of IFN responses in VA1-infected HIE and Caco-2 cells identified significant defects in the latter. Despite the strong IFN signature in HIE and the ability of exogenous type I and III IFNs to block HAstV infection, the endogenous IFN responses reduced, but failed to completely block, HAstV infection. Conversely, pharmacologic inhibition of IFN signaling further improved viral yields and in one case it overcame the lack of permissiveness of one HIE line. Collectively, this study provides fundamental insights into AstV biology in the intestinal epithelium and identifies HIE as a physiologically relevant pan-HAstV culture model.

## Results

### HIE from different intestinal segments support robust VA1 infection

**Fig 1:**
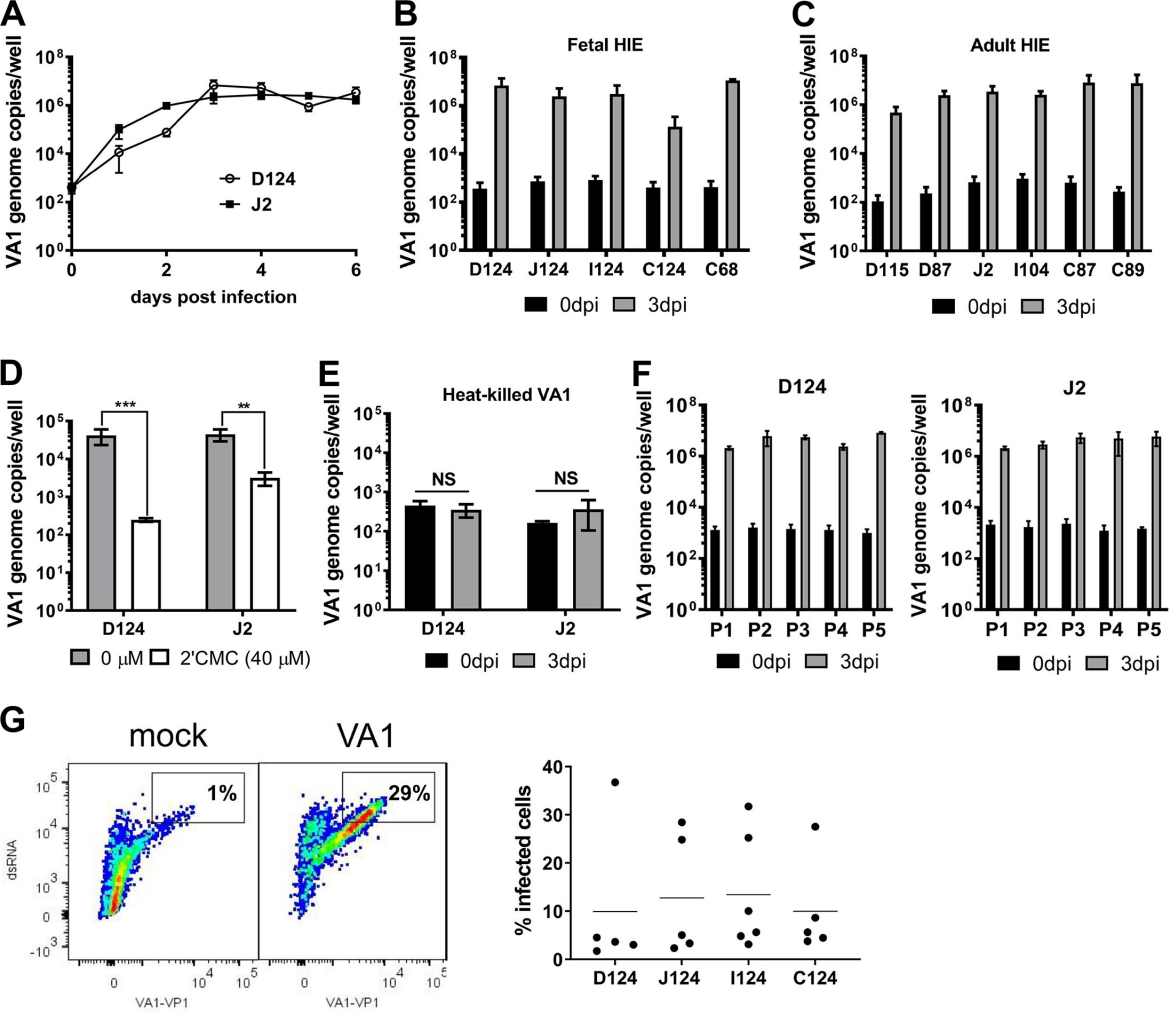
VA1 replicates in human intestinal enteroids (HIE). **A)** VA1 growth curve. Undifferentiated D124 and J2 HIE were cultured in 2D monolayers and infected with VA1 (MOI of 1). At indicated times, HIE were harvested and viral RNA was extracted and quantified by RT-qPCR. **B-C)** Undifferentiated HIE derived from indicated segments of fetal and adult intestines were infected with VA1 and analyzed as before. **D)** VA1 replication is blocked by the nucleoside analogue 2’-C-methylcytidine (2CMC). Undifferentiated D124 and J2 HIE were infected as before and cells treated with 40 μM 2CMC after adsorption. Cells were harvested and viral RNA quantified at 1 dpi. **E)** Heat-killed VA1 does not replicate in HIE. VA1 was heat-killed for 3 min at 100°C prior to infection of undifferentiated D124 and J2 HIE. Cells were harvested and viral RNA quantified at 3 dpi. **F)** VA1 remains infectious for 5 passages in HIE. Supernatant from infected D124 or J2 (passage 1, P1) containing ~10^3^ genome copies/ml was used to infect monolayers of D124 and J2, respectively. Cells were harvested and viral RNA quantified at 3 dpi. The procedure was repeated four times (P2-P5). For all experiments, viral genome copies were measured by RT-qPCR. **A–F)** Data are from ≥ 3 experiments; error = mean ± SD. **G)** A single cell suspension of VA1-infected (MOI of 1) D124, I124, J124 and C124 HIE was generated at 3 dpi and stained with antibodies against dsRNA and VA1 capsid protein prior to flow cytometry analysis. The left panel is a representative flow plot of mock- and VA1-infected J124 HIE. The right panel represents the percentage of double-stained cells in each segment. Each dot is a biological replicate. Abbreviations: dpi = days postinfection, D = duodenum, I = ileum, J = jejunum, C = colon. The numbers indicate patient identifiers. **P<0.01; ***P<0.001; NS = not significant.

VA1 is a non-canonical HAstV that can cause encephalitis in immunocompromised patients [16, 17], and it was the first non-canonical HAstV to be cultured [3]. It replicates in Caco-2 cells, HEK293, and A549 cells, but unlike classical HAstV, does not require trypsin activation [3]. To determine the susceptibility of HIE to VA1 infection, HIE derived from the fetal duodenum of patient #124 (D124) and adult jejunum of patient #2 (J2) were seeded in 2D monolayers and infected with a Caco-2 cell-derived stock of VA1 (MOI [multiplicity of infection] of 1). Cells were either frozen after adsorption (0 day post infection, dpi) or incubated at 37°C and harvested at selected dpi. VA1 infection kinetics were similar in adult J2 and fetal D124, with a 1–2 log_10_ increase in VA1 genome copies within the first 24 hours and a plateauing by 3 dpi (**Fig 1A**).

Since the replication kinetics indicated maximum viral titers by 3 dpi, we next investigated whether VA1 preferentially replicates in a specific region of the intestine or in a selected genetic background (i.e., patient donors) by comparing viral genome copies at 0 dpi vs 3 dpi. HIE lines derived from adult or fetal duodenum (D), jejunum (J), ileum (I) and colon (C) of the same patient (#124) or different patients (#2, #87, #89, #104, #115) were infected and VA1 viral yields were determined by RT-qPCR. Viral genome titers at 3 dpi revealed a consistent 2–4 log_10_ increase over 0 dpi regardless of the intestinal segment or donor analyzed (**Fig 1B and 1C**). The smallest increase was observed in fetal C124 HIE (~2 log_10_ fold increase). However, another fetal colon HIE line from a different donor (C68) supported ~4-log_10_ increase in VA1 replication. These data demonstrate all intestinal segments support VA1 infection, but the extent of replication may be donor-dependent.

To demonstrate that the increase in genome titers was due to active replication, HIE were treated after VA1 adsorption with the nucleoside analogue 2'-*C*-methylcytidine (2CMC) at the non-toxic concentration of 40 μM (**S1A Fig**). 2CMC is a polymerase inhibitor developed for hepatitis C virus but exhibits pan-antiviral activity against several RNA viruses, including nororvirus [18–21]. At 1dpi, VA1 infection was significantly reduced compared to vehicle control in both D124 and J2 HIE (**Fig 1D**). In addition, heat treatment (100°C, 3 min) of VA1 prior to infection of D124 and J2 HIE inhibited viral increases in genome titers at 3 dpi (**Fig 1E**). These data are indicative of active VA1 replication.

Next, we determined whether newly produced VA1 retained infectivity following HIE infection (**Fig 1F**). VA1-containing supernatants of D124 and J2-infected HIEs (~ 10^3^ genome copies) were serially passaged five times. A consistent 3–4 log_10_ increase in genome copies at 3 dpi was observed in both D124 and J2 HIE lines at each passage, demonstrating that HIE support production of infectious virions and thus support the complete viral life cycle.

Lastly, we measured the percentage of VA1-infected cells in the different intestinal segments from the same donor (D124, I124, J124 and C124) at 3 dpi (**Fig 1G, S1B Fig**). HIE were infected with VA1 (MOI = 1), and single cell suspensions were stained with antibodies against the virus capsid protein and double-stranded (ds) RNA, an intermediate of virus replication. Flow cytometry analysis revealed that the percentage of double-positive cells ranged between 2% and 37%. The largest variability in infection efficiency was observed in the duodenal segment (D124) despite detecting similar viral genome copies (**S1B Fig**). A comparison across different HIE segments showed no correlation between the percentage of infected cells and the corresponding amount of virus shed into the culture supernatant (S1C Fig). The reasons for this, including whether the observed differences are donor- (i.e. genotype-) specific, remain to be determined in future experiments.

Taken together, our data demonstrate VA1 replicates robustly in HIE from various segments of the intestine and from multiple adult and fetal patient donors. HIE support the full viral life cycle since HIE-derived virus can be serially passaged, and replication is blocked by addition of a polymerase inhibitor or by prior heat treatment of the virion.

### HIE differentiation status does not affect VA1 infection

**Fig 2:**
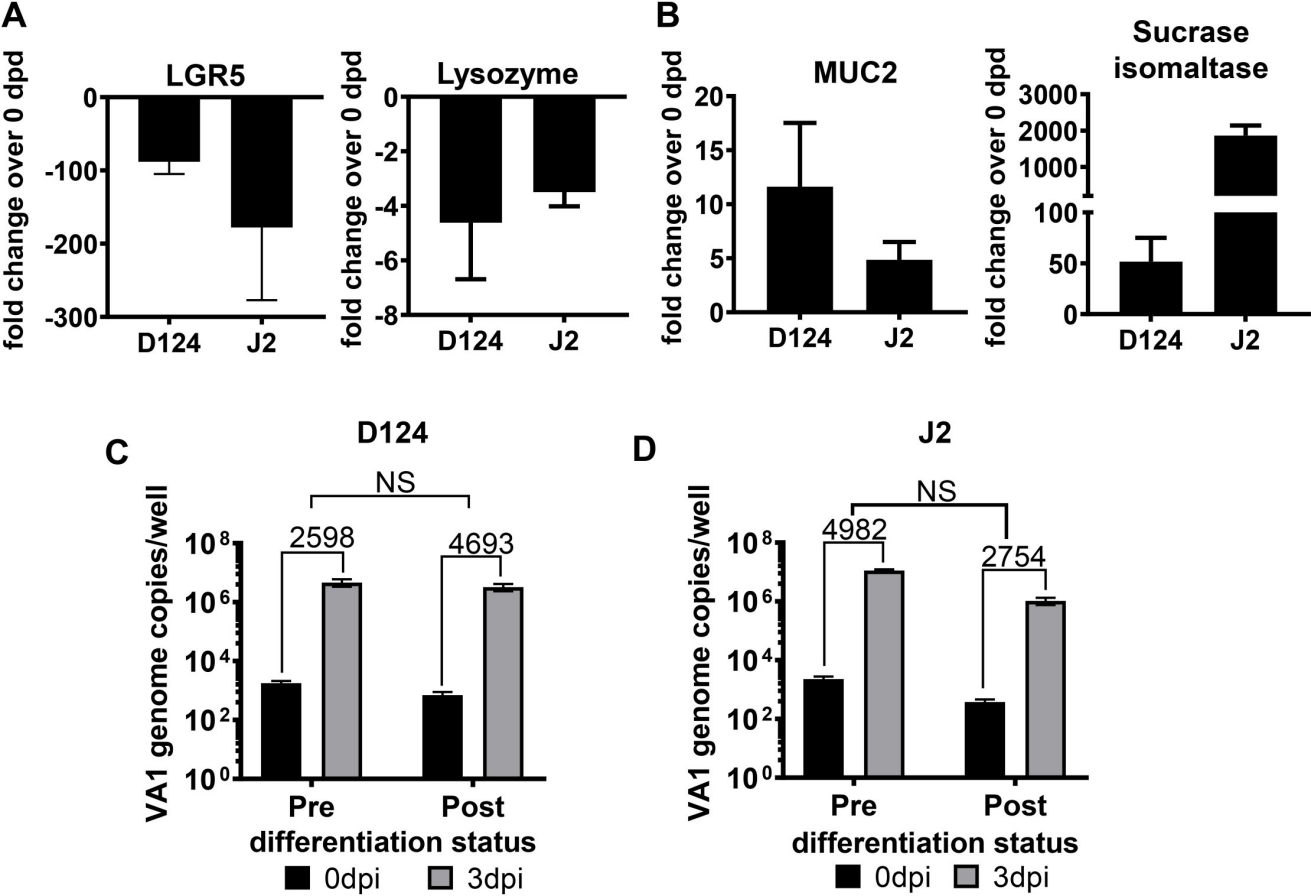
VA1 replication is not affected by the differentiation status of HIE. **A-B**) Differentiation status of J2 and D124 HIE monitored at 0 and 6 days post-differentiation (dpd) (after WNT3A removal) by measuring transcripts of (**A**) down-regulated genes—LGR5 (a stem-cell marker) and Lysozyme (a Paneth cells marker), and (**B**) up-regulated genes—MUC2 (a goblet cell marker) and sucrase isomaltase (a mature enterocyte marker). Transcript levels were measured by qPCR. Fold change is relative to GAPDH and is statistically significantly different (P<0.05) from the 0 dpd in **A)** and **B)**. **C-D)** VA1 replicates in HIE pre- and post- differentiation. **C**) D124 and **D**) J2 HIE were differentiated for 6 days, infected with VA1 (MOI of 1), and viral genome titers were measured at the indicated days by RT-qPCR. Fold increase was calculated by dividing 3 dpi virus titer with 0 dpi virus titer. N ≥ 3; error = mean ± SD. Abbreviations: D = duodenum, J = jejunum. The number associated with each letter indicates the patient identifier. The numbers on the bar chat indicate the fold increase in virus titer at 3 dpi over 0 dpi. NS = not significant.

Human norovirus (HuNoV) infects only differentiated epithelial cells [7], while human rotavirus replicates to higher titers in differentiated compared to undifferentiated HIE cultures [22]. Therefore, we compared the ability of VA1 to replicate in undifferentiated versus differentiated HIE. First, the differentiation status of HIE D124 and J2 lines was evaluated by comparing the transcripts of previously described marker-genes [7] at day 0 and 6 after withdrawal of WNT3A from the culture medium (days post-differentiation, dpd). As anticipated, transcripts for the stem cells markers LGR5 (**Fig 2A**) and olfactomedin 4 (OLFM4) (**S2A Fig**), and the Paneth cells marker lysozyme (**Fig 2A**) decreased during differentiation. Conversely, gene expression of mucin 2 (MUC2, a goblet cell marker) and the villus brush border enzyme sucrase-isomaltase (SI) increased over time (**Fig 2B**) together with intestinal alkaline phosphatase (IAP, a marker for terminally differentiated enterocytes), cytochrome P450 3A4 (a dominant drug-metabolizing enzyme in enterocytes), solute carrier family 15 member 1 (SLC15A1, an intestinal oligopeptide transporter) and solute carrier family 11 member 2 (SLC11A2, a divalent metal ion transporter) (**S2B Fig**). Similar trends in gene expression profiles were observed in D124 and J2 HIE, indicating that the adult or fetal origin or the segment-specificity did not affect differentiation *in vitro*. Next, to determine whether the HIE differentiation status modulated VA1 replication, D124 and J2 HIE at 0 (pre-) and 6 dpd (post-) were infected with VA1 at MOI of 1 and viral genome titers were determined by RT-qPCR. In contrast to norovirus and rotavirus, VA1 infected D124 (**Fig 2C**) and J2 (**Fig 2D**) HIE cultures to similar levels, irrespective of the HIE differentiation status. Furthermore, LGR5 and SI transcript levels were similar in both mock- and VA1-infected J2 culture at 3 dpi but different from mock-infected J2 three days after WNT3A withdrawal (**S2C Fig**). These data suggest that VA1 infection did not induce HIE differentiation. Taken together, these data demonstrate that HIEs from multiple intestinal segments can be successfully differentiated by withdrawal of WNT3A, but that the differentiation status of HIE does not alter susceptibility to VA1 and infection does not induce HIE differentiation.

### VA1 shows tropism for multiple cell types

**Fig 3:**
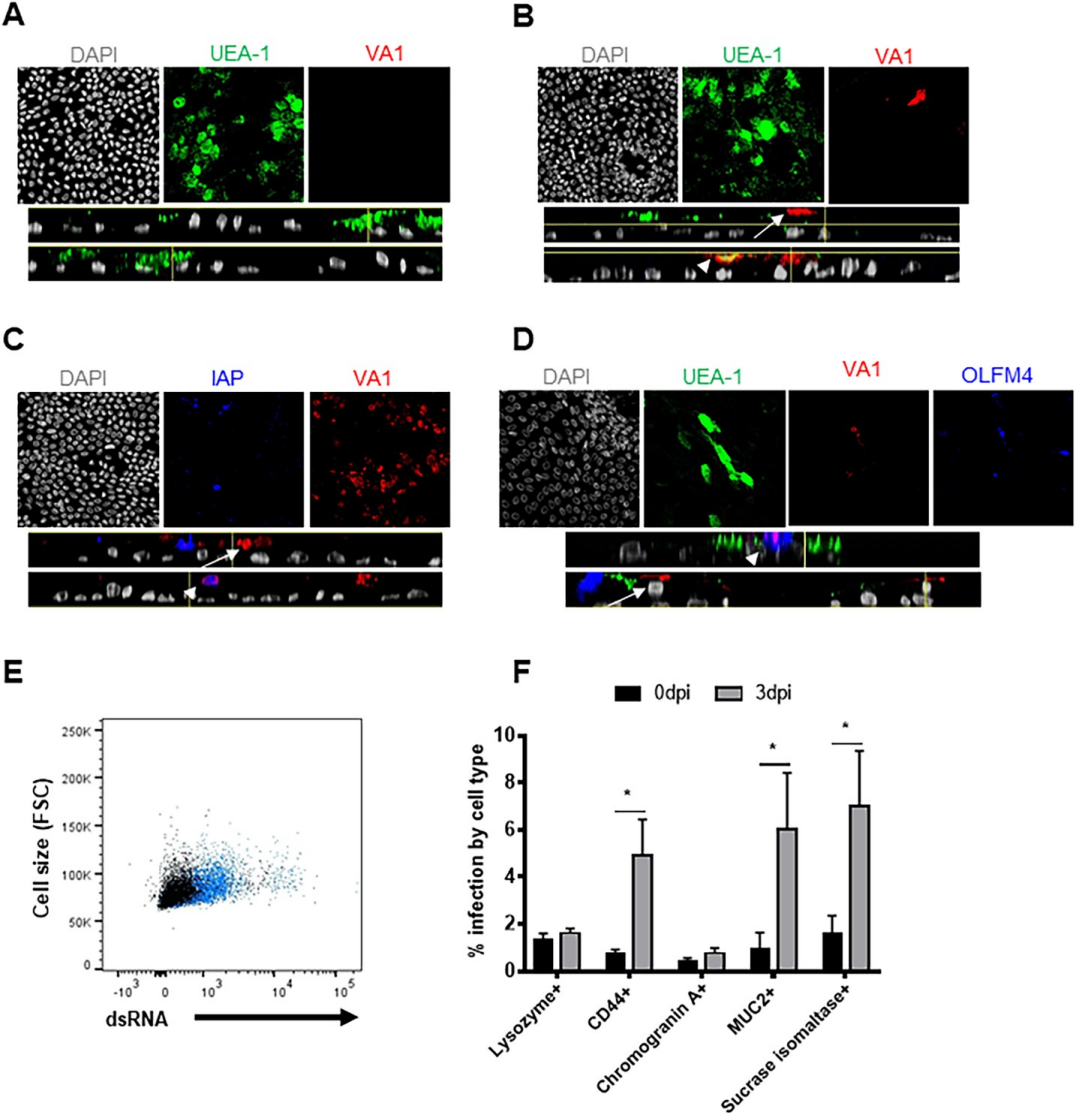
VA1 shows tropism for multiple cell types in HIE. **A-B)** VA1 replicates in UEA-1 positive and negative cells. D124 HIE were seeded in transwells, allowed to differentiate for 6 days and infected with **(A)** mock lysate or **(B)** with VA1 (MOI of 1). At 5 dpi, HIE were fixed with methanol and treated for immune fluorescence analysis. Virus was detected with a polyclonal mouse anti-VA1 serum followed by an anti-mouse AlexaFluor 594 (red) secondary antibody. Cells were also stained with the lectin UEA-1 conjugated with FITC (green), VA1-infected UEA-1 positive or negative cells are indicated with an arrowhead or arrow, respectively. **C)** VA1 infects intestinal alkaline phosphatase (IAP) positive and -negative cells. D124 HIE were seeded in transwells, allowed to differentiate for 6 days and infected with VAI (MOI of 1). At 5 dpi, HIE were fixed with methanol and treated for immune fluorescence analysis. Virus was detected with a polyclonal mouse anti-VA1 serum followed by an anti-mouse AlexaFluor 594 (red) secondary antibody. IAP was detected with an anti-IAP polyclonal antibody produced in rabbit and an anti-rabbit AlexaFluor 647 (blue). VA1-infected IAP -positive or -negative cells are represented with an arrowhead or arrow, respectively. D) VA1 infects OLFM4 positive and negative cells. I124 and J were seeded in transwells and allowed to differentiate for 6 days before infection with VAI (MOI of 1). At 3 dpi, cells were harvested and fixed for immunofluorescence analysis with a polyclonal mouse anti-VA1 serum followed by an anti-mouse AlexaFluor 594 (red); anti-OLFM4 antibody produced in rabbit and anti-rabbit AlexaFluor 647 (blue) and the UEA-1 lectin (green). VA1-infected OLFM4 -positive or -negative cells are represented with an arrowhead or arrow, respectively. All the images were acquired with a Nikon A1 laser confocal microscope and analyzed with Fiji Image J software. Images were representative of an N = 4 (UEA-1) or N = 2 (IAP and OLFM4) experiments. Planar view of Mock or VA1-infected HIE is represented in the upper panels with the single channels hyperstack projected on a max intensity Z-plane in the lower panels with side XY views of the hyperstack. **E-F)** VA1 infects sucrase isomaltase (SI)-, mucin 2 (MUC2)- or CD44-positive cells. Differentiated I124 HIE were infected with VA1 (MOI of 1) and a single cell suspension was obtained at 3 dpi. Cells were stained with the surface markers lysozyme (paneth cells), CD44 (progenitor cells), chromogranin A (enteroendocrine cells), MUC2 (goblet cells) and sucrase isomaltase (mature enterocytes) and the intracellular dsRNA antibody conjugated with biotin followed by the secondary streptavidin-APC Cy7 antibody. **E)** Representative flow plot of VA1-infected cells (blue) vs mock-infected cells (black)**. F)** The percent of infected cells in specific cell sub-populations. N = 2 biological replicates, each containing n = 3 technical replicates. The Mann-Whitney U test was used to analyze the difference between the percent positive cell types in VA1-infected cells at 0 vs 3 dpi. *P<0.05.

Classical HAstV was shown to replicate in mature enterocytes of the human small intestine [23]. However, the tropism of the non-canonical HAstVs remains unknown. Intriguingly, VA1 replicated similarly in HIE regardless of the differentiation status (**Fig 2C and 2D**), although different cell types are enriched in undifferentiated vs. differentiated cultures [15]. Therefore, we set out to determine the VA1-infected cell type(s) in HIE. D124 HIE were seeded on transwells as 2D monolayers and differentiated for 6 days by withdrawal of WNT3A. Differentiation of polarized monolayers was verified by trans-epithelial electrical resistance (TEER) measurement, whereby all monolayers exhibited values ≥ 400 Ω/cm^2^. D124 HIE were then infected with VA1 (MOI of 1) for 5 days, fixed and processed for immunofluorescence staining. Cells were stained with an anti-VA1 mouse immune serum and UEA-1 (*Ulex europeus* agglutinin 1), a lectin that binds α-1,2-fucose residues. Mock-infected cells served as negative controls. UEA-1 lectin was chosen as it can stain multiple intestinal cell types (e.g., entero-endocrine cells [24], colon adenocarcinoma cells [24], small intestinal epithelial cells [25], goblet cells [26], Paneth cells [27]) in different mammalian species. As anticipated, no viral staining was observed in mock-infected cells (**Fig 3A**). Intriguingly, viral antigen staining was observed in UEA-1-positive cells as well as in UEA-1-negative cells (**Fig 3B**). To corroborate the observation that VA1 may infect multiple cell types, additional immunofluorescence experiments were performed. Differentiated VA1-infected D124 monolayers were stained with anti-VA1 serum and an anti-intestinal alkaline phosphatase (IAP; marker for terminally differentiated enterocytes) antibody, while differentiated VA1-infected I124 monolayers were stained with anti-VA1 serum, UEA-1 and an anti-OLFM4 (a stem cell marker [28]) antibody. Interestingly, VA1 antigen staining was observed in both IAP-positive and IAP-negative cells (**Fig 3C**), as well as in OLFM4-positive and OLFM4-negative cells (**Fig 3D**). To quantify the infection in different cell types and independently confirm the multicellular tropism of VA1 in HIE, we used a complementary flow cytometry approach. Unfortunately, in our hands the same antibodies did not work for both approaches. Thus, differentiated VA1-infected I124 HIE cells were stained with antibodies to cell surface markers SI, MUC2, lysozyme, CD44 (marker of progenitor cells [29]) or chromogranin A (ChrA, for enteroendocrine cells), and with a biotin-conjugated anti-dsRNA antibody for virus detection. An overall increase of dsRNA staining was observed in VA1-infected over mock-infected HIE (~5% of dsRNA-positive cells, **Fig 3E**), consistent with our previous observations (**Fig 1G**). The dsRNA-positive population was detected in SI-, CD44-, and MUC2-positive cells, but not in lysozyme- or ChrA-positive cells (**Fig 3F, S3 Fig**). Taken together, these data indicate that VA1 has a tropism for multiple cell types in HIE, including progenitor cells, adsorptive enterocytes, and likely also goblet cells.

### VA1 infection elicits time-dependent transcriptional responses in HIE

**Fig 4:**
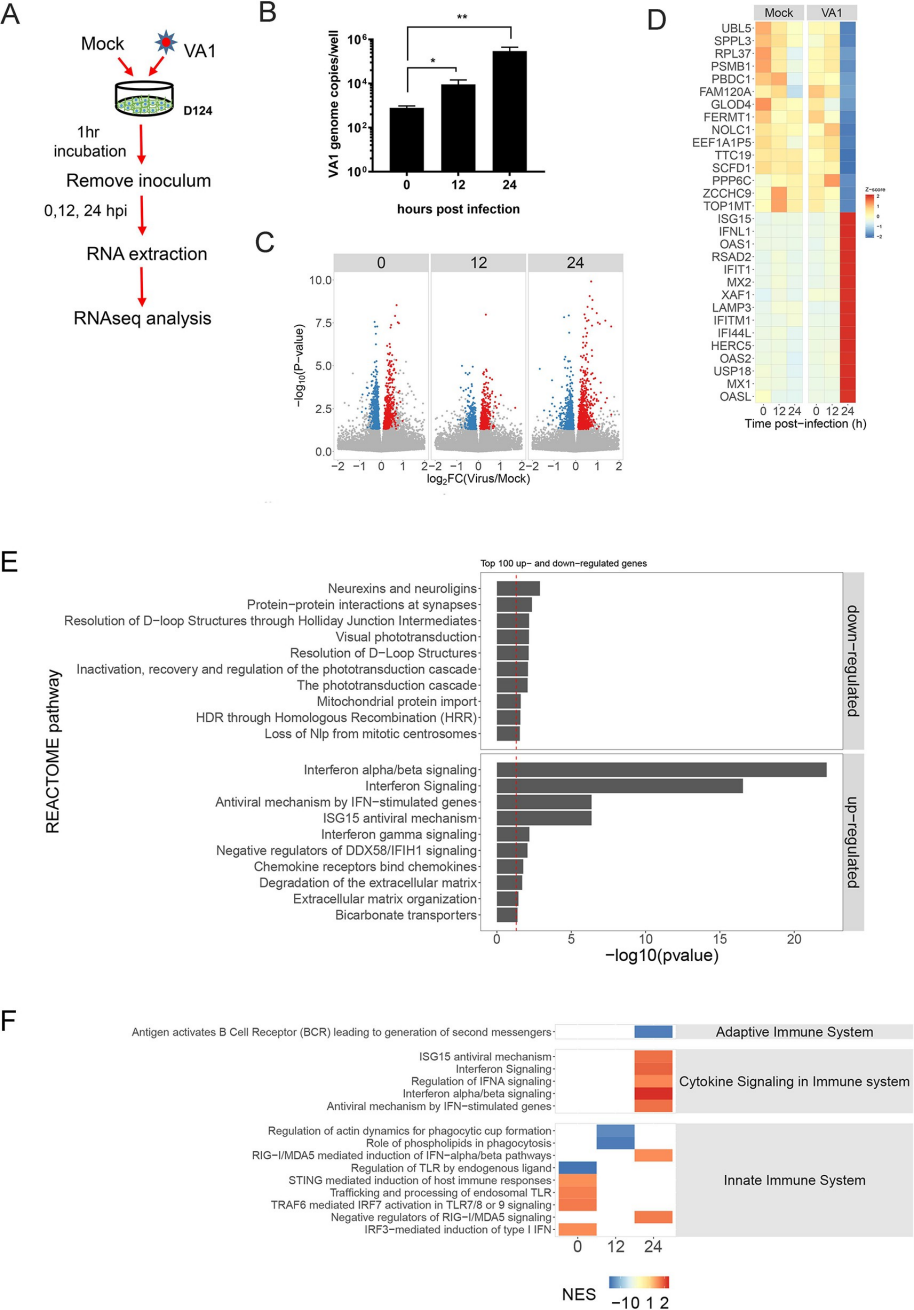
Transcriptional profiling of VA-1 infected HIE by RNA-seq. **A)** Experimental design of the RNA-seq experiment. N = 3 for each time point. **B)** VA1 titers of infected undifferentiated D124 HIE measured by RT-qPCR at the indicated times. *P<0.05; **P<0.01. **C-D)** Host response to VA1 infection by RNA-seq. **C)** Log_2_ fold change in normalized expression (transcripts per million reads) of all expressed host genes of VA1-infected HIEs relative to mock-infected HIEs at 0, 12, and 24 hpi (Volcano plot). The y-axis shows the -log_10_ transformed P-value. Genes significantly up-regulated (P < 0.05) in VA1-infected HIEs relative to mock-infected HIEs are colored red and significantly down-regulated genes are colored blue. **D)** Heat map of the top 15 up- and down-regulated genes (Z-score) in VA1-infected HIEs relative to mock-infected HIEs at indicated time points post-infection. **E)** The pool of genes that were significantly up- or down-regulated in VA1-infected HIEs relative to mock-infected HIEs were evaluated for enrichment of REACTOME pathway analysis using an over-representation test. The top 10 significantly over-represented pathways among both the up- and down-regulated gene sets are shown. **F)** Differentially expressed genes were ranked according to the log_2_ fold change between VA1-infected HIEs and mock-infected HIEs and analyzed for coordinated gene expressed within REACTOME pathways using Gene Set Enrichment Analysis. The heat map shows conditions in which the normalized enrichment score (NES) differs significantly (P < 0.05) from random variation, indicating a trend towards coordinated up- or down-regulated expression of genes within a pathway.

The host response of the primary intestinal epithelium to HAstV infection has not been elucidated to date. In order to characterize the global effects of AstV infection on human intestinal tissue, we performed transcriptional profiling of VA1-infected HIE by RNA-seq. We used the D124 line for our studies since AstV infections are typically symptomatic in very young children [2], and our prior infection studies indicated rapid infection in D124 (**Fig 1A, 1B and 1F**). D124 HIE were mock-infected or infected with VA1 (MOI = 1) and harvested at 0 hpi (i.e., 1h post-adsorption), 12 hpi, and 24 hpi (**Fig 4A**). To monitor viral replication, VA1 genome copies were detected by RT-qPCR, revealing an approximately 1 and 2 log_10_ increase at 12 and 24 hpi, respectively (**Fig 4B**). Total RNA was extracted and analyzed by RNA-seq. VA1 genome reads contributed 0.05 ± 0.02% of total RNA-seq reads at 24 hpi. Genome copies as determined by RT-qPCR were correlated to the proportion of viral transcripts in the pool of sequenced RNA collected from the same HIE cultures, revealing an exceptionally strong correlation between the two measures of viral replication (r^2^ = 0.98, *P* = 2.5 x 10^−14^; **S4A Fig**). Differential expression analysis was performed to identify genes associated with the HIE host response to VA1 infection. A total of 23,220 genes were detected. To visualize the scale of the transcriptional response to viral infection, genes differentially expressed at each time point were graphed in a volcano plot (**Fig 4C**). The log_2_ fold change in normalized expression (transcripts per million reads [TPM]) of all expressed host genes in VA1-infected HIEs relative to mock-infected HIE is shown on the x-axis, while the -log_10_ transformed P-value is given on the y-axis. Genes that are significantly up-regulated (P < 0.05) prior to the application of multiple testing corrections in VA1-infected D124 HIE relative to mock-infected HIE are colored red, while significantly down-regulated genes are colored blue. In addition, we also determined the number of genes that are significantly different after multiple testing corrections were applied (**S1 Table**). Even after applying this more conservative approach, 110 genes were significantly upregulated and 136 genes were downregulated after a 1 hour adsorption (0 hpi), indicating changes due to viral attachment to cells. This number was reduced at 12 hpi, with 8 significantly upregulated and 5 downregulated genes. At 24 hpi, 154 upregulated and 49 downregulated genes compared to the mock-infected control were identified.

We next identified the top 15 significantly up- and down-regulated genes at 24 hpi. This group of genes was used to generate a heat map of the mean scaled fold-change (Z-score) in expression of each of them in virus-infected HIE relative to mock-infected HIE at each time point (**Fig 4D**). Most of the upregulated genes at 24 hpi were involved in type I and type III interferon (IFN) signaling. Of the IFN genes, *IFNL1* was highly upregulated, with *IFNA1* and *IFNB1* upregulation being slightly lower (**S4B Fig**). No upregulation was observed for the genes encoding IFN-γ, or the type I and III IFN receptors (**S2 Table**). The top 12 IFN-stimulated genes (ISGs) also positively correlated with VA1 infection (**S4C Fig**). Conversely, the top 12 downregulated genes, including fermitin family member 1 (FERMT1), signal peptide peptidase like 3 (SPPL3), and tetratricopeptide repeat domain 19 (TTC19), negatively correlated with VA1 infection (**S4D Fig**).

Next, we evaluated lists of the top 100 up- and down-regulated genes at 24 hpi using an over-abundance test to identify significantly over-represented REACTOME pathways in these lists. These data revealed that the top four significantly enriched pathways among upregulated genes were all related to innate antiviral signaling (**Fig 4E**), which will be investigated in more detail below. For downregulated genes, the top two pathways were “neurexins and neuroligins”, which play signaling roles in synapse development [30], and “protein-protein interactions at synapses”. The biological significance of synapses during astrovirus infection remains to be elucidated in the future.

In order to evaluate the potential for coordinated and directional activation of genes in known signaling pathways, we applied gene set enrichment analysis (GSEA) to our RNA-seq differential expression data. Based on the strong dominance of IFN signaling pathways, we focused our GSEA analysis on immune signaling (**Fig 4F**). During the adsorption phase, nucleic acid pattern recognition receptor signaling pathways (TLRs, STING) were upregulated, consistent with their early role in virus recognition and induction of IFN signaling. At 24 hpi, these early signaling events had been largely replaced by the later phase of IFN signaling and expression of ISGs.

Taken together, these data indicate that VA1 infection predominantly elicited antiviral IFN signaling in the HIE-derived fetal duodenum at the transcript level.

### HIE, but not Caco-2 cells, mount strong type I and III IFN responses to HAstV infection

**Fig 5:**
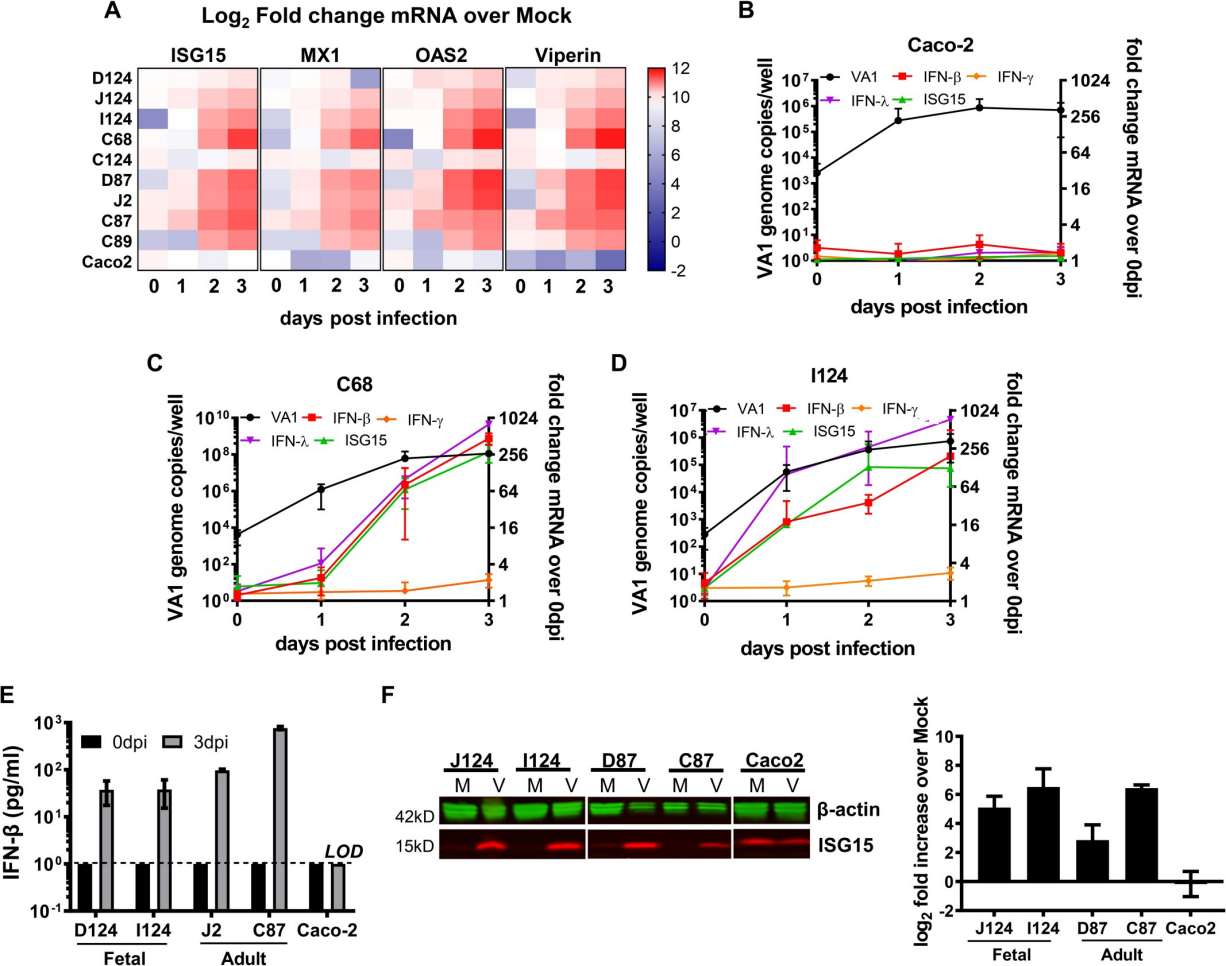
HIEs mount interferon responses to VA1 infection. **A)** Heat map of ISG15, MX1, OAS2 and Viperin (RSAD2) mRNA fold increase over mock during VA1 infection of HIE derived from different human intestinal segments and Caco-2 cells. **B-D)** Timecourse of IFN-β, IFN-γ, IFN-λ and ISG15 mRNA expression in VA1 infected Caco-2 cells (**B**), C68 HIE (**C**), and I124 HIE (**D**). VA1 infected cells were harvested at selected dpi and cellular RNA was extracted for VA1 quantification by RT-qPCR (left y-axis) and for the remaining transcripts quantification as fold increase over 0 dpi by qPCR (right y-axis). GADPH was used as internal control. **E)** IFN-β is secreted during VA1 infection in HIE but not in Caco-2 cells. D124, I124, D87, J2 and Caco-2 cells were infected with VA1 or mock infected (MOI of 1). Supernatants were collected at 3 dpi and ELISA was used to measure IFN- β. **F)** Mock and VA1-infected HIE and Caco-2 cells lysates were collected at 3 dpi. ISG15 protein levels were analyzed and quantified by Western blot using the secondary LI-COR fluorescent antibodies. Βeta-actin was used as internal control. Images were acquired with LI-COR Odyssey Imager and quantified with ImageJ software for the densitometry analysis. Representative blots are shown on the left for fetal (J124 and I124) and adult (D87 and C87) VA1 infected HIE. Quantification of the data is shown on the right. N ≥ 3; error = mean ± SD. Abbreviations: D = duodenum, J = jejunum, I = ileum, C = colon, M = mock infection, V = VA1 infection. The numbers indicate patient identifiers. *P<0.05; ***P<0.001; ND = not detected; LOD = limit of detection.

The dominant transcriptional response in VA1-infected D124 HIE was related to IFN signaling (**Fig 4**). To confirm and expand on our RNA-seq findings, we selected four ISGs among the top 15 upregulated genes; interferon-stimulated gene 15 (ISG15), 2’-5’-oligoadenylate synthetase 2 (OAS2), myxovirus resistance 1 (MX1), and radical s-adenosyl methionine domain-containing protein 2 (RSAD2; viperin) and evaluated transcriptional changes in response to VA1 infection in multiple HIEs (D124, J124, I124, C68, C124, D87, J2, C87, and C89) and Caco-2 cells by RT-qPCR (**Fig 5A**). The inclusion of multiple fetal and adult HIE lines is aimed at investigating the translatability of findings across host genotypes. Caco-2 cells were included in the analysis as these immortalized cells have traditionally been used for studies of HAstV innate immunity [31, 32]. The log_2_-fold increases in ISG transcripts of VA1-infected HIE and Caco-2 cells over mock are shown in a heat block map (**Fig 5A**). Increases of mRNA transcripts were typically observed over the timecourse of infection in the different intestinal segments with consistently low transcript levels at 1 dpi and significant increases by 3 dpi. Interestingly, transcript levels of IFNs and ISGs did not increase in Caco-2 cells despite the ~ 2 log_10_ increase in VA1 genome titers (**Fig 5A bottom row, Fig 5B, S3 Table**). These data point to differences in innate immune signaling in Caco-2 cells. On the other hand, C68 (**Fig 5C**) and I124 (**Fig 5D**; **S4 Table**) HIE responded with increases in transcript levels of IFN-β, IFN-λ, and ISG15 to VA1 infection over the 3 day timecourse. As anticipated for epithelial cells, no changes in IFN-γ transcript levels were observed in HIE or Caco-2 cells (**Fig 5B–5D**; **S3 and** S4 Tables). However, the kinetics of the IFN response and ISG induction varied between different HIE lines (**Fig 5A, 5C and 5D**). Overall, the RT-qPCR data confirmed the general trends observed in the RNA-seq dataset.

Surprisingly, at 1 dpi, in spite of the >2 log_10_ increase in VA1 genome titers, there were very minimal increases in IFN-β and ISG15 transcript levels in the C68 HIE (**Fig 5C**). To test whether this HIE line was capable of mounting an IFN response at this early time point (i.e., 1 dpi), we stimulated C68 HIE with polyinosinic:polycytidylic acid (poly I:C), a synthetic dsRNA [33], or infected them with Sindbis virus (MOI = 1) and vesicular stomatitis virus (VSV, MOI = 1), two viruses known to strongly induce IFN responses [34] (**S5A Fig**). In contrast to VA1-infected C68 HIE, Sindbis virus infection and poly I:C treatment strongly upregulated ISG15 gene expression in C68 HIE at day 1 post infection/treatment. VSV infection induced ISG15 gene expression to a lesser degree despite higher VSV titer compared to Sindbis virus (**S5A and S5B Fig**). This suggested that the low ISG response observed in the context of VA1 infection in C68 HIE is virus-specific and that this HIE line is capable of a robust antiviral response within 24 hours.

To confirm that transcriptional changes also affected protein expression, we next measured IFN-β secretion by cytokine ELISA in supernatants collected from VA1-infected HIE derived from different intestinal segments and patients (D124, I124, J2 and C87) (**Fig 5E)**. While no IFN-β protein was detected at the start of infection (0 dpi) in all HIE tested, significant amounts were detected at 3 dpi with the D124 and I124 supernatants containing approximately 50 pg/mL IFN-β, J2 supernatants ~100 pg/mL, and C87 supernatants ~1000 pg/mL. Consistent with the lack of increases in IFN-β transcript levels, no IFN-β protein was detectable in VA1-infected Caco-2 supernatants (**Fig 5E**).

In addition to IFN secretion, we also evaluated the expression of ISG15 protein by Western blot in HIE following VA1 infection. Our data showed that at the protein level, ISG15 is over-expressed in VA1-infected HIE J124, I124, D87 and C87 at 3 dpi as compared to mock-infected HIE, consistent with the transcript data (**Fig 5F**). Consistent with the lack of induction of ISG15 transcript levels, no increase in ISG15 protein was detected in Caco-2 cells (**Fig 5F**). Intriguingly, there was a high baseline level of ISG15 protein in mock-infected Caco-2 cells, further highlighting the dysregulation of antiviral signaling in this transformed cell line.

Taken together, these data confirmed the induction of a strong antiviral IFN response in multiple HIE lines at the transcriptional and protein level upon VA1 infection. Caco-2 cells, on the other hand, exhibited dysregulated IFN signaling responses, a finding common to many transformed cell lines [35]. Regardless of the strength of the antiviral IFN response, VA-1 replicated robustly in either culture system.

### VA1 is sensitive to exogenous IFNs

**Fig 6:**
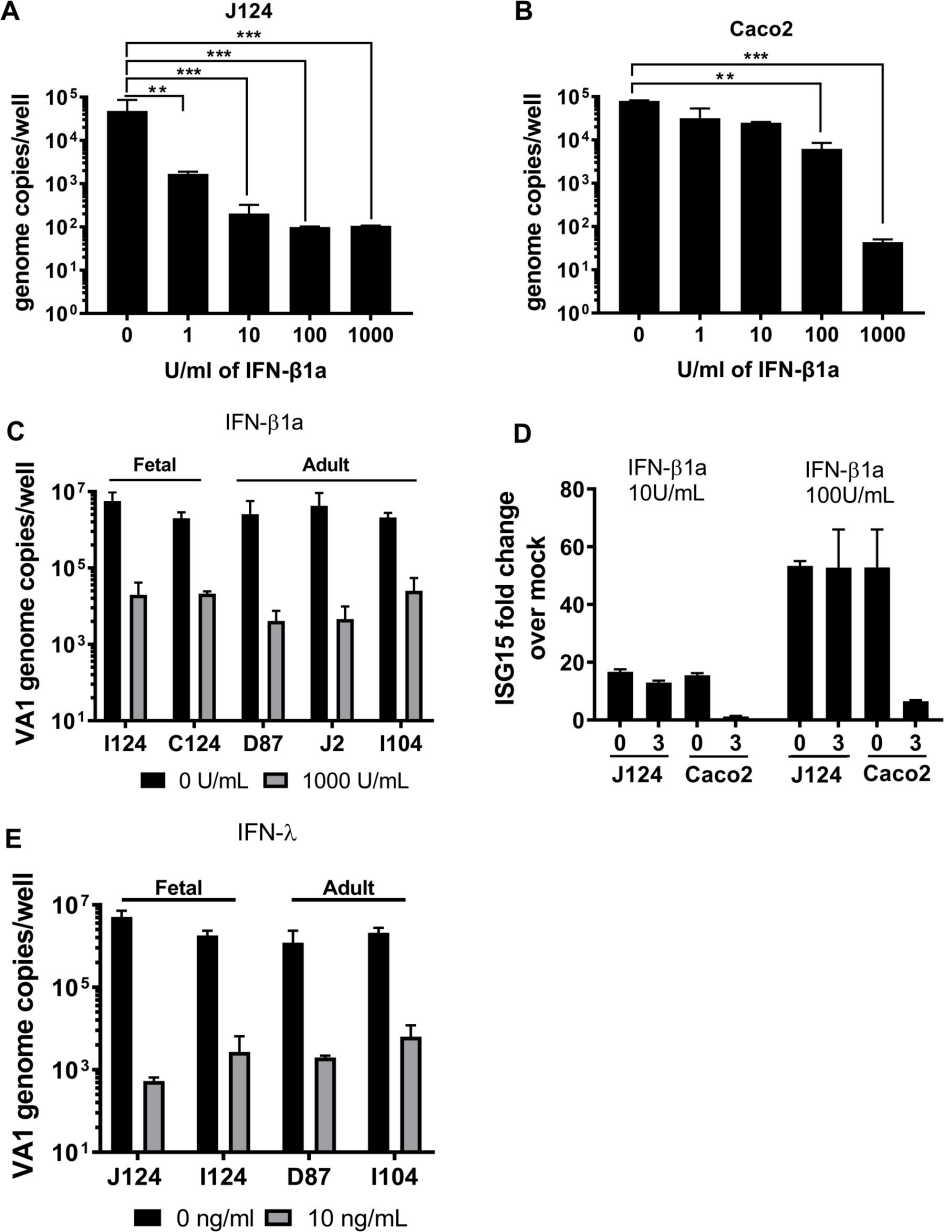
Astrovirus is sensitive to exogenous interferon. **A)** J124 HIE and **B)** Caco-2 cells were treated with increasing concentrations of IFN-β1a 12 hrs before VA1 infection (MOI of 1). Viral genome copies were measured by RT-qPCR from extracted cellular RNA at 3 dpi. **C)** VA1 sensitivity to exogenous IFN-β in HIE derived from different human intestinal segments. Fetal (I124 and C124) and adult (D87, J2 and I104) HIE were treated with IFN-β1a (1000 U/ml) 12 hrs before VA1 infection. VA1 genome copies were measured by RT-qPCR at 3 dpi. **D)** J124 and Caco-2 cells were treated with 0, 10, and 100 U/ml IFN-β 12 hrs before VA1 infection. ISG15 transcript levels in VA1-infected HIE or Caco-2 cells were measured at 0 dpi and at 3 dpi. The ISG15 fold increase was calculated comparing to 0 U/ml IFN-β treated cells after normalizing to GAPDH. **E)** J124, I124, D87 and I104 HIE were pre-treated with IFN-λ (10 ng/ml) for 12 hrs before VA1 infection (MOI of 1). Viral genome copies were measured by RT-qPCR from extracted RNA at 3 dpi. Data are from ≥ 3 experiments; error = mean ± SD. Abbreviations: D = duodenum, J = jejunum, IFN = interferon. The numbers indicate patient identifiers. *P<0.05; **P<0.01; ***P<0.001.

As we observed robust VA1 infection of HIE in the presence of a strong IFN response (**Fig 5**), we hypothesized that VA1 may be insensitive to the antiviral activity of IFNs. Hence, increasing concentrations of IFN-β were exogenously added to J124 HIE or Caco-2 cells 12 hours before infection with VA1 (MOI of 1). A concentration-dependent reduction of VA1 genome copies was detected in both J124- and Caco-2-infected cells by RT-qPCR (**Fig 6A and 6B**). Notably, IFN-β treatment was more efficient in J124 than Caco-2 cells (EC_90_ = 0.17 U/ml vs. 632.65 U/ml). To expand this finding to other intestinal segments, we next treated I124, C124, D87 and J2 HIE with 1000 U/ml IFN-β 12 hours before VA1 infection. Exogenous IFN-β significantly reduced virus titer in all infected HIE segments tested (**Fig 6C**), confirming that VA1 is sensitive to type I IFNs. To confirm the activity of IFN-β, we also quantified ISG15 transcript levels in J124 and Caco-2 cells by RT-qPCR **(Fig 6D**). Prior to VA1 infection (i.e., 12 hours post IFN treatment and 0 dpi), ISG transcript levels were significantly increased in IFN-β-treated as compared to non-treated control. However, at 3 dpi ISG15 transcripts were significantly lower in Caco-2 cells at all concentrations compared to 0 dpi, further pointing to dysregulated IFN responses in these cells. On the other hand, HIE were able to synthesize IFN and maintain robust IFN signaling in response to VA1 infection throughout the 3 day infection.

Type III IFN controls viral infections at the intestinal epithelial barrier [36] and the genes encoding IFN-λ were strongly upregulated in our RNA-seq dataset. Therefore, to evaluate the antiviral efficacy of type III IFN responses against VA1, J124, I124, D87 and I104 HIE were treated with IFN-λ (10 ng/ml) prior to infection and during infection **(Fig 6E)**. Similar to IFN-β treatment, VA1 infection was also sensitive to exogenous type III interferon treatment with > 3 log_10_ decreases in genome copy number at 3 dpi compared to the non-treated control (0 U/ml). Taken together, these data demonstrate that VA1 is sensitive to exogenous type I and type III IFNs. However, the dose-response curves of the antiviral activity of type I IFNs differ between transformed Caco-2 cells and non-transformed HIE cultures.

### VA1 replication is limited by endogenous IFN responses

**Fig 7:**
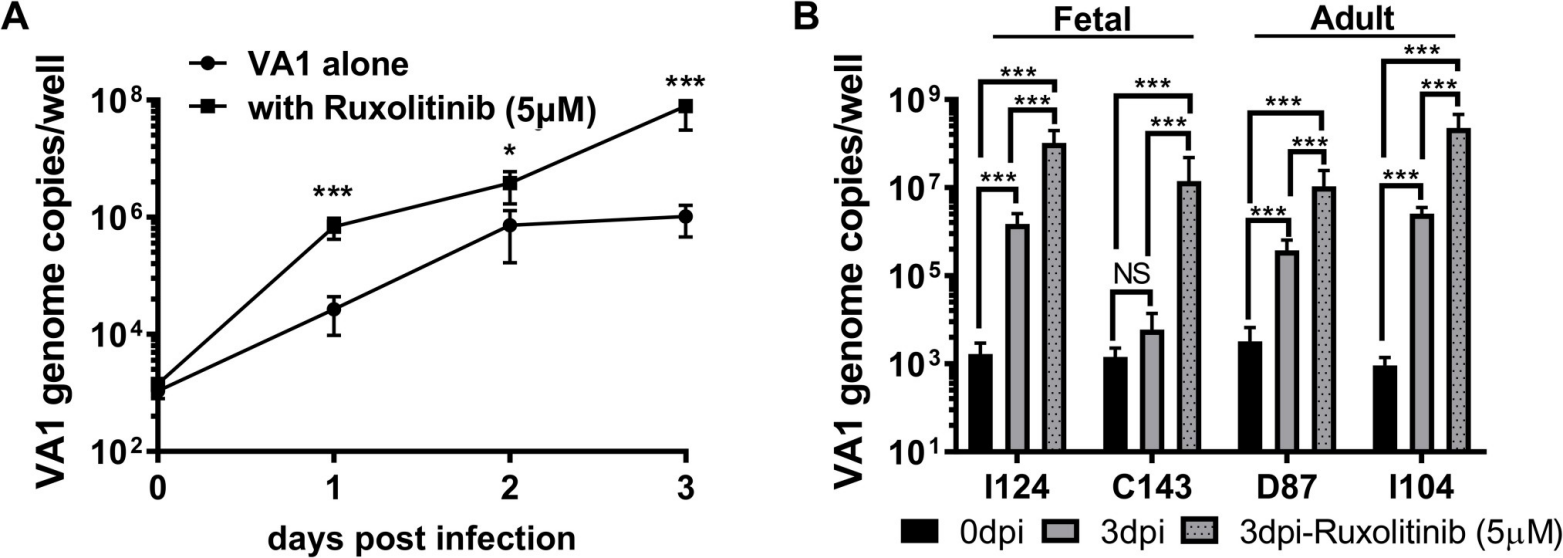
Inhibition of endogenous interferon responses enhances VA1 infection. **A)** Timecourse of VA-1 infection in the presence or absence of ruxolitinib. C68 HIE was treated with ruxolitinib (5 μM) for 12 hrs before VA1 (MOI = 1) infection. Viral genome copies were determined by RT-qPCR from extracted RNA at days 0, 1, 2 and 3 post infection. **B)** The effect of ruxolitinib (5 μM) treatment on VA1 infection of selected HIE derived from different human intestinal segments was determined at 3 dpi by RT-qPCR. Data are from ≥ 3 experiments; error = mean ± SD. Abbreviations: D = duodenum, I = ileum, C = colon. The numbers indicate patient identifiers. NS = not significant, ***P<0.001.

Since VA1 infection is sensitive to exogenous addition of type I and III IFNs, we next questioned whether the endogenous IFN response limited VA1 infection. Towards that end, we used ruxolitinib, an ATP mimetic janus-associated kinases (JAKs) inhibitor that blocks STAT1 activation and inhibits ISG induction [37, 38]. C68 HIE were pre-treated with ruxolitinib (5 μM) 12 h before VA1 infection (MOI of 1). Cells were harvested at selected dpi for RT-qPCR analysis (**Fig 7A**). Remarkably, ruxolitinib treatment resulted in increased VA1 replication already at 1 dpi with peaks of 10^8^ genome copies/well reached by 3 dpi. Increased replication was also observed upon ruxolitinib treatment of VA1-infected I124, C143, D87 and I104 HIEs (**Fig 7B**). While all HIE lines showed some increase in VA1 genome copies with ruxolitinib treatment, pharmacologic inhibition of IFN signaling had the greatest effect on VA1 infection in C143 HIE, which could not be infected in the absence of the inhibitor. This finding pointed to differences in the strength of the endogenous IFN response in different HIE lines and highlighted the importance of validating findings across different donor genotypes. Collectively, these data indicate that VA1 is also sensitive to endogenous IFNs, but that the IFN response is unable to completely block viral replication in all HIE.

### All HAstV clades infect HIE but replication is controlled by endogenous IFN responses

**Fig 8:**
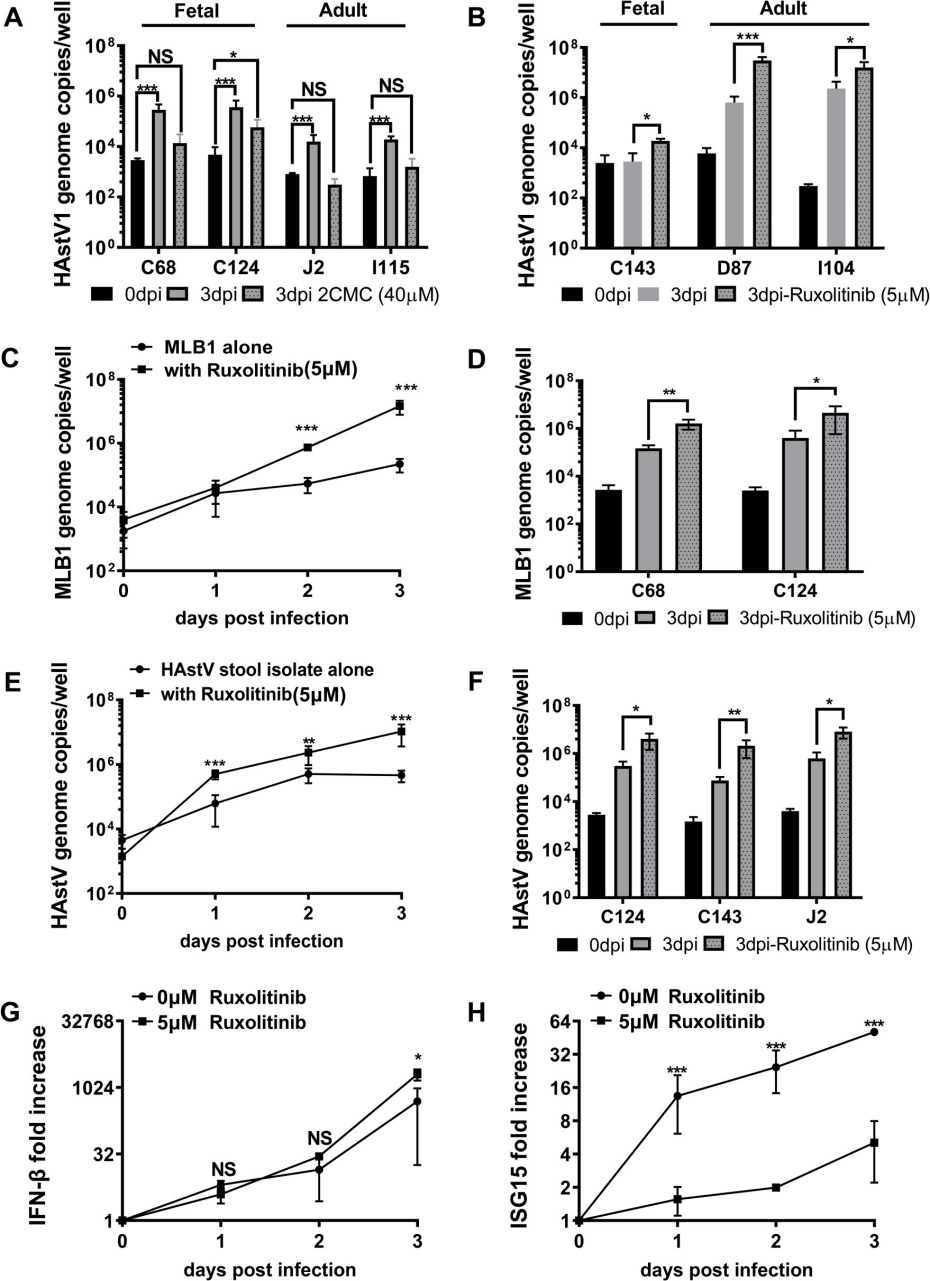
Pharmacological blockage of endogenous IFN response increases the infection of HIE by HAstV from all clades. **A)** HAstV1 (MOI = 1) infection of selected HIE derived from different human intestinal segments was carried out in the presence of trypsin and, when indicated, cells were further treated with 40 μM 2’-C-methylcytidine (2CMC) after adsorption. Viral genome copies were determined at 0 and 3 dpi by RT-qPCR. **B)** HAstV1 (MOI = 1) infection of selected HIE derived from different human intestinal segments was carried out in the presence of trypsin and pre-treated for 12 hrs with or without ruxotinilib (5 μM). Viral genome copies were determined at 0 and 3 dpi by RT-qPCR. **C)** Timecourse of MLB1 (MOI = 1) infection of C143 HIE in the presence or absence of ruxotinilib (5 μM) treatment. HIE was treated with ruxolitinib (5 μM) for 12 hrs before astrovirus infection. Virus genome copies were determined by RT-qPCR from extracted RNA at days 0, 1, 2 and 3 post infection. **D)** MLB1 (MOI = 1) infection of selected HIE pre-treated for 12 hrs with or without ruxotinilib (5 μM) was measured at 0 and 3 dpi. **E)** Timecourse of stool-derived HAstV (MOI = 1) infection of C68 HIE was carried out as described for HAstV-1. **F)** Infection of indicated HIE with stool-derived HAstV (MOI = 1) for 0 vs. 3 dpi as before. **G-H)** Effect of ruxotinilib (5 μM) treatment on **G**) IFN-β and **H**) ISG15 transcript expression in C68 HIE infected with stool-derived HAstV. Data are from ≥ 3 experiments; error = mean ± SD. Abbreviations: D = duodenum, J = jejunum, I = ileum, C = colon, IFN = interferon. The numbers indicate patient identifiers.

To expand our findings to other HAstV strains, we next tested the susceptibility of HIE to representative members of the two other HAstV clades and a clinical stool sample containing HAstV as well as their sensitivity to ruxolitinib. As a representative of the classical HAstV clade, we obtained HAstV-1 grown in Caco-2 cells in the presence of trypsin, since it has been studied extensively over the last few decades [39–41]. HIE from multiple donors and intestinal segments were infected with HAstV-1 (MOI = 1) for 3 days and viral genome titers were determined by RT-qPCR. HAstV-1 replicated in all four HIE lines tested (C68, C124, J2, I115) in the presence of trypsin (**Fig 8A**). Increases in viral genome copies were sensitive to 2CMC treatment, confirming productive replication. To determine whether endogenous IFN responses limit HAstV-1 replication in HIE, similar to our findings with VA1, we infected three additional HIE lines (C143, D87, I104) with HAstV-1 in the presence or absence of ruxotinilib (5 μM) (**Fig 8B**). To our surprise, HAstV-1 only replicated in two of the three HIE lines tested. Similar to VA1 infection, no increase in genome copies over the 3 days infection was observed in C143 HIE, but infection was rescued in C143 HIE by pharmacologic inhibition of IFN signaling with ruxolitinib. In addition, ruxolitinib further increased genome titers in the other two HIE lines, demonstrating that HAstV-1 is sensitive to endogenous IFN signaling and that the genetic background of the host (i.e., patient donor) influences the ability of HAstV to replicate in these non-transformed cells.

Next, we tested a representative of the MLB clade. Towards that end, we used a MLB1 stock grown in Huh7.5 cells [5] and infected three HIE lines (C68, C124, C143). MLB1 infections were performed similar to those with VA1 above in the absence of trypsin. MLB1 replicated efficiently in all HIE tested, including the VA1 and HAstV1 refractive C143 HIE (**Fig 8C and 8D**). Treatment with ruxolitinib further increased viral genome copies, demonstrating MLB1 is also sensitive to the endogenous IFN response in HIE.

Lastly, we tested the ability of HIE to support replication of HAstV directly from a stool sample (**Fig 8E and 8F**). A de-identified clinical stool sample containing HAstV was obtained from the University of Michigan Health system. The sample amplified with primers to HAstV, but not MLB- or VA-specific primers. Given the trypsin dependence of classical HAstV, the clinical isolate was trypsin treated prior to and during the infection. The virus strain replicated efficiently in all four HIE lines (C68, C124, C143, J2) tested (**Fig 8E and 8F)**. Interestingly, this included the C143 HIE line that was only permissive to VA1 and HAstV-1 in the presence of ruxolitinib. As observed with the other strains, ruxolitinib treatment also enhanced replication of the clinical isolate in HIE (**Fig 8F)**. Analysis of IFN-β and ISG15 transcript levels during infection in the presence and absence of ruxolitinib confirmed the ability of the drug to inhibit IFN signaling and ISG induction, but not the induction of IFN itself (**Fig 8G and 8H**).

Together, these data demonstrate that HAstV strains from all three clades and a clinical stool sample were amenable to replication in HIE from different intestinal segments and genetic backgrounds. However, the trypsin requirement of these viruses differed with the classical HAstV strains, but not the non-classical ones, requiring trypsin activation for successful infection of HIE. Nevertheless, HIE responded similarly to all HAstV infections by induction of an IFN response and infections were enhanced in the presence of inhibitors of IFN signaling. Thus, the innate antiviral response limits, but does not eliminate, infection by members of all three HAstV clades.

## Discussion

Astroviruses (AstV) are a largely understudied class of highly prevalent enteric viruses. Here we report, for the first time, that human intestinal enteroids (HIE) from multiple intestinal segments and donors are susceptible to infection by all clades of HAstV. We identified progenitor cells, goblet cells and enterocytes as cell types infected with HAstV-VA1 in HIE. In addition, we determined that the host response to HAstV infection in HIE is predominated by a type I and III interferon (IFN) response. However, typically this response only restricts but does not prevent HAstV replication in HIE.

The lack of a physiologically relevant cell culture system for all HAstV clades has been a limiting factor for studying many aspects of HAstV biology and has prevented comparative studies. Classic HAstV replication was reported in several continuous cell lines [6], including Caco-2 cells [42]. HAstV-VA1 also successfully replicates in Caco-2 cells [3], whereas HAstV-MLB1 and HAstV-MLB2 were recently reported to infect Huh7.5 cells, Huh7AI, and A549 cells, but not Caco-2 cells [5]. Although lung epithelial A549 cells are susceptible to some classical HAstV serotypes [6], HAstV-VA1 [3], and HAstV-MLB1/2 [5], no intestinal epithelial cell line can be infected with HAstV from all clades. Our study has overcome this limitation by demonstrating that HIE support replication of members from all three clades. Previous studies revealed that trypsin aids AstV infection *in vivo* by cleaving the virus capsid [43], and in the case of classic HAstV, trypsin treatment is required for *in vitro* replication in Caco-2 cells. Our experiments in HIE confirmed that trypsin is indeed required for infectivity of classic HAstV, but not for the VA or MLB strains tested. Interestingly, epithelial cells of the gastrointestinal tract express high levels of trypsin [44]. This may explain why the classical HAstV infections are generally limited to the gastrointestinal tract when infecting immunocompetent hosts [2]. In contrast, evidence of non-canonical HAstVs, belonging to the VA and MLB clades, spreading beyond the gastrointestinal tract has been reported [45]. HAstV-MLB2 has been associated with the upper respiratory infection of a child [46, 47] and CNS disease, while HAstV-VA has been associated with celiac disease [48] and encephalitis [49]. Interestingly, our observation regarding the ability of VA1 and MLB1 to replicate in HIE without addition of trypsin may correlate with their propensity for systemic spread, a hypothesis that requires future investigations.

HIE provide an *in vitro* model that recapitulates intestinal morphology and physiology [50] and has been used to reveal new aspects of rotavirus [51], norovirus [7], enterovirus [52] and adenovirus [53] infection in intestinal epithelial cells. In our hands, HAstV-VA1 robustly replicates in both differentiated and undifferentiated HIE, in contrast to rotavirus and norovirus that preferentially or exclusively infect differentiated HIE, respectively [7]. The ability to infect HIE of varying differentiation status suggested a broader cell tropism and led us to investigate the cells type(s) infected by VA1. Initial immunofluorescence analysis of UEA-1 stained monolayers pointed to a tropism for multiple cell types because VA1 infection was detected in both UAE-1- positive and negative cells. The UEA-1 lectin is expressed on a variety of cell types [54]. To identify the infected cell types, we performed additional immunofluorescence microscopy and flow cytometry on VA1-infected cells. Despite the technical difficulties in identifying suitable antibodies for both methodologies, both approaches detected viral antigen in cells expressing markers for mature enterocytes (i.e., IAP and sucrase isomaltase), and CD44- and OLFM4-positive cells, which are markers for progenitor cells or stem cells, respectively. Flow cytometry further indicated viral replication in MUC2-positive cells, which is a marker for goblet cells. Identification of at least three different cell types infected by VA1 that are enriched either in undifferentiated HIE cultures (CD44- and OLFM4-positive cells) or differentiated cultures (goblet cells and enterocytes) also provided an explanation of our finding that HIE were susceptible to VA1 infection irrespective of the differentiation status. Understanding viral tropism can shed light on viral pathogenesis. *In vivo*, analysis of a rare small intestinal biopsy sample from an immunocompromised child infected with classical HAstV demonstrated viral antigen in epithelial cells near the villus tips of the jejunum and duodenum, while paracrystalline viral arrays were detected in enterocytes by electron microsopy [23]. Data from classical HAstV infection in gnotobiotic lambs also point to mature enterocytes in villi of the small intestine as the main infected cell type [55, 56]. Our finding of enterocyte infection by VA1 *in vitro* points to a shared tropism of this member of the VA clade with other AstV. Surprisingly, VA1 further infected CD44- or OLFM4-positive progenitor cells and goblet cells in HIE cultures. Other enteric viruses appear to have a narrower tropism. Human noro-virus infects mature enterocytes [7], while adenovirus preferentially infects goblet cells [53]. Human rotavirus infects both enterocytes and enteroendocrine cells [22], a tropism hypothesized to be critical for induction of the vomiting response [57]. However, VA1 is the first enteric virus with a tropism for CD44- and OLFM4-positive cells. Whether the broader tropism of VA1 for intestinal (our study and [3]) and neural cells [4] underlies the observed extraintestinal spread, and whether the tropism for multiple cell types is matched by other HAstVs remains to be determined in future studies.

Large gaps exist in our collective understanding about the host response to HAstV infections. Virus infection, including replication of several enteric viruses, rotavirus [58], reovirus [59] and norovirus [60], is sensitive to the antiviral function of type I and/or III IFN. Previous *in vitro* reports also demonstrated the sensitivity of AstV infection to innate immune responses. Specifically, HAstV replication induces type I interferon (IFN) responses later during infection *in vitro* [61], and pre-treatment of Caco-2 cells with type I IFN reduces viral genome loads, viral protein synthesis, and the virus-induced barrier permeability [32]. Conversely, neutralization of type I IFNs or inhibition of the Tank-binding kinase 1 (TBK1), a mediator in the IFN signaling cascade, increased viral titers. Similar to HAstV-1 [32], VA1 is also sensitive to type I IFNs in Caco-2 cells *in vitro* [3], and MLB HAstVs are sensitive to type I and III IFN in Huh7 cells [5]. Our findings confirm the antiviral activity of type I and III IFNs on VA1 infectivity in HIE. Furthermore, our RNA-seq data showed strong induction of type I and type III IFNs, but not type II IFN, in response to AstV infection of fetal duodenum HIE, resulting in a dominant antiviral ISG signature. This finding was confirmed in multiple HIE from various segments and donors, pointing to a uniform host response to VA1 infection by the human epithelium. This response is shared with AstV in other species. For example, murine AstV infection induces IFN-λ in intestinal epithelial cells, which depending on the mouse background, can protect the animals from subsequent murine norovirus or murine rotavirus infection [62]. Interestingly, while this response is shared with influenza virus, a respiratory virus, which also induces both type I and III IFNs in primary lung epithelial cells [63, 64], it is different from rotavirus, which upregulated only type III, but not type I IFNs, in HIE [65, 66]. The endogenous IFN response to rotavirus infection in HIE was not sensitive to anti-IFN blocking antibodies, leading the authors to question whether type III IFN induction plays an antiviral role against HRV in HIE [65]. Similarly, blocking type III IFN responses during reovirus infection only increased reovirus transcript levels two-fold [67]. In our study, pharmacologically blocking the JAK1/STAT pathway [38] and the downstream antiviral ISG pathway resulted in 1–3 log_10_ increases in AstV infection of HIE. More importantly, virus infection with strains from all three clades was enhanced by this treatment. These data demonstrate a role for endogenous IFN responses in curtailing AstV replication in this non-transformed cell system despite the generally robust viral loads. This partial control of HAstV infection by innate immune responses point to a physiological response of HIE to viral infection similar to what one would expect *in vivo* where epithelial innate response synergistically acts with mucosal immunity (and at later times post infection with adaptive immunity) to systemically control and ultimately clear the infection. It is remarkable that we were able to study this intricate interplay between innate immunity responses and viral replication in the HIE model, providing a foundation for more detailed studies of these responses in the future.

Our comparison of innate immune signaling in HIE with Caco-2 cells, also highlighted HIE as a more physiologically relevant cell culture model of intestinal epithelial cells. Previous studies of other human viruses have also uncovered differences in infection parameters between established cell lines and primary non-transformed human cells [68, 69]. In our study, transformed Caco-2 cells did not show increases IFN or ISG transcript or protein production during HAstV-VA1 infection. Similarly, neither MLB AstVs, nor HAstV-1 induced strong IFN expression in infected cell lines [5, 32]. This points to defects in the innate immune sensing and/or signaling in these transformed cell lines. Collectively, these observations highlight the limitations of studying virus-host interactions in immortalized cell lines, in particular with regards to innate antiviral responses.

Taken together, our findings highlight for the first time the utility of HIE as a single replication system for a wide range of HAstV strains, likely including clinical isolates, and thus a valuable model to study HAstV biology. They also identify intestinal progenitor cells as a previously unrecognized target cell type for an enteric virus. Furthermore, our data also validate HAstV as a model enteric RNA virus. First, HAstVs infect HIE regardless of the differentiation status, intestinal region, or the genetic background of the donor. Second, they typically replicated to higher viral loads in HIE compared to the other previously investigated enteric viruses. Third, the host response observed against HAstV has many common features to other enteric virus infection: i.e. rotaviruses, reoviruses, noroviruses, enterovirus 71, parvovirus, and porcine epidemic diarrhea virus, which were all shown to be induced and/or to be controlled by IFN [65]. In addition, our comparative studies of antiviral immune responses in non-transformed HIE and transformed Caco-2 cells underline the importance of studying host responses in non-transformed cell systems. Collectively, establishment of the HIE system for AstV research represents a technical breakthrough for the astrovirus field and lays the foundation for a broad range of basic and translational discoveries in the future.

## Materials and methods

### Cells and virus stocks

Caco-2 human colon adenocarcinoma cells (ATCC), African green monkey kidney Vero cell (ATCC) and Huh7.5 cells [70] were maintained in Dulbecco’s modified Eagle’s medium (Gibco) supplemented with 10% fetal bovine serum, 1% penicillin/streptomycin, 1% L-glutamine, 1% non-essential amino acids, and 1% HEPES (Gibco). Cells were maintained at 37°C with 5% CO_2_ and grown to about 90% confluence before virus infection. Human intestinal enteroids (HIE) derived from fetal or adult duodenum, jejunum, ileum and colon were obtained from the University of Michigan Medical School Translational Tissue Modeling Laboratory. The adult jejunum J2 line was kindly provided by Dr. M. Estes (Baylor College of Medicine). A summary of all the HIE lines used in the study and characteristics are listed in **S5 Table**. 3D Enteroids were derived as previously described [71–73], suspended in Matrigel (Corning) and maintained in complete L-WRN medium [74]. The Complete L-WRN medium contained 50% WRN conditioned media (ATCC), Advanced DMEM/F-12 (Gibco), 2mM GlutaMax (Invitrogen), 10mM HEPES (Invitrogen), 1% penicillin/streptomycin (Gibco), 1X N-2 media supplement (Invitrogen), 1X B-27 supplement minus vitamin A (Invitrogen), 100 μg/ml Primocin (InvivoGen), 1 mM N-Acetyl-L-cysteine (Sigma-Aldrich), 50 ng/ml EGF (Invitrogen), 10 μM SB202190 (Sigma-Aldrich), 500 nM A83-01 (Tocris), 10 mM nicotinamide (Sigma-Aldrich), 10 nM [Leu15]-Gastrin I (Sigma-Aldrich) and 10μM Y27632 (R&D Systems). For 2D monolayers, HIE were dissociated into a small cell aggregates and seeded in transwells or 48 well plates coated with human collagen type IV as described [15]. All infections were performed on 2D monolayers. HAstV-1 and HAstV-VA1 were expanded in Caco-2 cells, while HAstV-MLB1 was expanded in Huh7.5 cells to create a virus stock. A P3 stock was used for all experiments. The clinical stool isolate was collected in AMES buffer and filtered through a 0.22 μm filter before direct infection of HIE. VSV and Sindbis virus were propagated and plaqued on Vero cells as described [75]. For viral infections, MOI are based on plaque-forming units for VSV and Sindbis, and genome titers for the different HAstVs.

### Antibodies: Generation of an anti-VA1 antibody

Swiss Webster mice were purchased from Charles River Breeding Laboratory. A concentrated VA1 virus stock was generated as described previously for murine norovirus [76]. Each mouse was then infected orally and intraperitoneally with 100 μl each of concentrated VA1 and reinfected with the same dose 14 days later. Mice were sacrificed and blood collected by cardiac puncture. Blood was then centrifuged at 3000 x g for 15 min and serum was stored at -20°C. ELISA was used to confirm the reactivity of the mouse serum to VA1 as previously described [77].

### Generation of anti-VA capsid antibody

The VA1 capsid sequence was analyzed using OptimumAntigen (Genscript) to identify antigenic regions, and an optimal peptide sequence was selected from amino acid positions 532–545 (YP_003090288.1) for antibody production. A cysteine residue was added to the N-terminus to facilitate conjugation (CNSEEWHTNAEQPHQ). The peptide was commercially produced and inoculated into rabbits by the vendor (Genscript). Pre-immune sera and affinity purified antibody (WAB111; 1.32 mg/mL) from sera 35 days post-inoculation were collected.

Commercial antibodies used in this study are listed in **S6 Table**.

### Ethics statement

Studies were performed according to local and federal guidelines as outlined in the 'Guide for the Care and Use of Laboratory Animals' of the National Institute of Health. Protocol 00008736 was approved by the University of Michigan Committee on Use and Care of Animals.

### Cell viability assay

HIE were treated with increasing concentrations of 2CMC for three days to determine toxicity [78]. Cell viability was then tested using WST-1 assay (Roche) according to the manufacturer’s recommendations.

### RNA isolation

RNA for RT-qPCR was extracted using the Direct-zol RNA MiniPrep Plus (Zymo Research) according to the manufacturer’s directions. One step qRT-PCR was used to quantify AstV genome copies as described [3]. Two step RT-PCR was used to quantify relative expressions of ISGs as described [79]. GAPDH was used to normalize gene expression. All primers used in this study are listed in **S7 Table**.

### Viral infections

Briefly, 1x10^5^ HIE were seeded in 48-well plates as 2D monolayers and incubated overnight at 37°C with 5% CO_2_ in complete L-WRN medium. Undifferentiated HIE were maintained in complete medium and allowed to form a confluent 2D cell monolayer, while differentiated HIE were cultured for 6 days in differentiation medium (Complete L-WRN media without WRN, nicotinamide, SB and 5% Noggin). Next, HIE were inoculated with AstV for 1 hour at 37°C and washed once with culture medium without growth factors. Fresh complete L-WRN medium was then added. Samples for 0 dpi were harvested and frozen immediately. All remaining samples were incubated at 37°C with 5% CO_2_ and harvested at the indicated time point. For HAstV-1 and filtered AstV-positive clinical stool sample infection, virus was treated with 10 μg/ml porcine trypsin (Sigma-Aldrich) for 30 min at 37°C before inoculating HIE for 1 hour in media containing 5 μg/ml trypsin. VA1 and MLB1 infections required no trypsin treatment. All infections were performed at MOI of 1 based on genome copies per cell. In some cases, HIE were exposed to a non-toxic concentration of 2CMC (40 μM) for the length of viral infection assay as described [78] to confirm virus replication. For IFN treatments, HIE were treated with 10-fold serial dilutions of 1000 U/ml IFN-β1a or 10 ng/ml IFN-λ (both PBL Assay Science) 12 h prior to astrovirus infection as described [3]. In case of ruxolitinib treatment, HIE were treated with 5 μM ruxolitinib (Cayman Chemical Company) 12 h prior to astrovirus infection. Infected cells were incubated in media containing the desired drug concentrations for the duration of viral infection as described [3].

### Immunofluorescence

F124 HIE were seeded on transwells and allowed to differentiate for 6 days by WNT3A withdrawal prior to infection with VA1 (MOI of 1). At 5 dpi, infected HIE were fixed with 4% paraformaldehyde (PFA) and permeabilized with 0.1% Triton X-100 for 20 min at room temperature. After a one hour blocking period with PBS + 2% BSA, HIE were stained with i) anti-rabbit ZO1 primary antibody and the mouse anti-VA1 polyclonal serum for 1h at room temperature, ii) secondary anti-rabbit AlexaFluor 594, anti-mouse AlexaFluor 647, or *Ulex europeus* agglutinin 1 (UEA-1) lectin conjugated with FITC for 1h at room temperature, and finally iii) with 4′,6-diamidino-2-phenylindole (DAPI) for 10 minutes. After 5 washes with PBS + 2% BSA, the transwell membranes were cut out and mounted on slides with ProLong antifade (Invitrogen). Images were captured using a Nikon inverted laser scanning confocal microscope. Image analysis was performed by using Fiji.

### Flow cytometry

At 3 dpi, HIE monolayers were harvested and separated into a single cell suspension by using Accumax (STEMCELL technologies, USA). HIE were kept in Advanced DMEM/F-12 (Gibco) supplemented with 2 mM of EDTA and sequentially stained with LIVE/DEAD Fixable Aqua Dead Cell Stain (Thermo Fisher Scientific) and (when indicated) with surface markers: CD44-BV421, Mucin-2-FITC, Sucrose-isomaltase-PE, CGA-PerCP and Lysozyme-APC. Cells were next fixed with Fixation/Permeabilization Concentrate (eBioscience) and kept in permeabilization buffer for intracellular staining either with i) the biotin-conjugated primary dsRNA (J2, Scicons) followed by the secondary streptavidin APC-Cy7-antibody, or ii) the primary antibodies dsRNA (J2, Scicons) and VA1-VP1 followed by the secondary anti-mouse AlexaFluor 488 and anti-rabbit AlexaFluor 564, respectively. For the surface marker, single color controls were obtained by staining BJAB B cells with CD45 labelled with BV421, FITC, PE, PerCP and APC whereas the intracellular staining was based on VA1-infected Caco-2 cells. Samples were acquired with BD Fortessa and analysed by FlowJo. For infection studies, the gate on the mock-infected control samples was set to ≤1% of positive events. Gates for the surface markers were set using undifferentiated HIE data as reference.

### ELISA

IFN-β secretion was determined in HIE supernatants using VeriKine human IFN-β ELISA kit (PBL Assay Science) according to the manufacturer’s protocol.

### Protein extraction, SDS-PAGE and immunoblotting

HIE and Caco-2 cells were washed with cold DPBS++ (Dulbecco's Phosphate-Buffered Saline with calcium and magnesium) before lysis. Protein was extracted from virus or mock-infected cell lysate, separated by SDS-PAGE and transferred to Immobilon-FL transfer membranes (IPFL00010 Pore size 0.45 μm) as described [80]. The membrane was blocked in PBS + 0.05% Tween-20 + 5% BSA at 4°C overnight and probed with a rabbit anti-ISG15 or mouse anti-β-actin antibody at 4°C overnight. Secondary LI-COR fluorescent antibodies were added for 1.5 hours at room temperature and then visualized using the LI-COR Odyssey Imager. ImageJ was used to quantify the Western blots by densitometry and normalizing bands to β-actin.

### RNA Sequencing

RNA was isolated using the mirVana RNA isolation kit and following the Total RNA isolation protocol (Thermo-Fisher Scientific, Waltham MA). RNA library preparation and RNA-sequencing (single-end, 50 bp read length) were performed by the University of Michigan DNA Sequencing Core using the Illumina Hi-Seq 2500 platform. All sequences were deposited in the EMBL-EBI ArrayExpress database using Annotare 2.0 and are cataloged under the accession number E-MTAB-8527.

### RNA-seq alignment

Pseudoalignment of raw Illumina sequence reads was computed using kallisto v0.44.0[81]. All sequences were aligned to *Homo sapiens* Genome Reference Consortium human build 38 release 79 (GRCh38.rel79) and the astrovirus VA1 genome (GenBank: 4731478). Aligned reads against each genome were tabulated separately.

### RNA-seq quantification and differential expression analysis

Differential expression of pseudoaligned sequences was calculated using the R package DEseq2[82]. The multiple testing-adjusted p-value was calculated using the DESeq2 implementation of the Wald test[82] to compare viral-infected HIEs to mock-infected HIEs by time point.

### RNA-seq gene set enrichment analysis

Gene pathway over-representation tests and Gene Set Enrichment Analysis[83] were implemented using the R packages clusterProfiler[84] and ReactomePA[85]. Conserved gene pathways were retrieved from the Gene Ontology Consortium (GO) database[86] and REACTOME database[87].

### Statistical analyses

All RNA-seq analysis was conducted in R (*R Core Team*, *R*: *A language and environment for statistical computing (R Foundation for Statistical Computing*, *Vienna*, *Austria*, *2017; https://www.R-project.org/)*.) using GNU Emacs v25.1[88] on the 64-bit Debian Linux stable version 9 operating system. Plots were constructed using the R package ggplot2 (*H*. *Wickham*, *Ggplot2*: *Elegant graphics for data analysis (Springer-Verlag New York*, *2009;* *http*:*//ggplot2*.*org*). Data analysis scripts and further documentation for the RNA-seq analysis are available https://github.com/hilldr/astrovirus.

The statistical analyses for the remaining experiments were performed using Prism software, version 7 (GraphPad Software, CA). Student t test, or ANOVA was used to determine statistical significance as appropriate.

## Supporting information

S1 Fig **A) 2CMC is non-toxic in HIE at working concentration**. Undifferentiated J2 HIE were treated with increasing concentrations of 2’-C-methylcytidine (2CMC) for 3 days (duration of VA1 infection) and cell viability was determined by WST1 assay (Roche). B) **VA1 infects HIE from different compartments**. A single cell suspension of VA1-infected (MOI of 1) D124, I124, J124 and C124 HIE was obtained at 3 dpi. After fixation/permeabilization, cells were stained with antibodies against dsRNA and VA1 capsid protein and analyzed by flow cytometry. Left panel represents the % of double-positive cells. On the right, the supernatants of infected HIE were harvested and RNA was extracted for RT-qPCR analysis. **C) Virus genome copies and % of infected cells do not correlate.** Analysis of the data represented in B and linear regression (95% confidence interval).
(TIF)

S2 Fig Changes in transcript levels of select genes in undifferentiated vs. differentiated HIE. The differentiation status of J2 and D124 HIE was monitored at 0 and 6 days post-differentiation (dpd) (after WNT3A removal) by measuring transcripts of **A)** olfactomedin 4 (OLFM4), a stem cell marker, and **B)** alkaline phosphatase, cytochrome P450, SLC15A1, and SLC11A2 (markers of enterocytes). **C)** differentiation status of J2 HIE monitored at 0 and 3 days post differentiation after WNT3A removal (CMGF-) or VA1 infection without removal of WNT3A (CMGF+). Transcript levels were measured by qPCR. Data are from ≥ 3 experiments; error = mean ± SD. Fold change is relative to GAPDH, and is statistically significantly different (P<0.05) from the 0 dpd in **A)** and **B)**. In **C)** ***P<0.001. Abbreviations: D = duodenum, J = jejunum. The number associated with each letter indicates the patient identifier. NS = not significant.
(TIF)

S3 Fig **Representative flow plots of the percent of VA1-infected cells (red) vs mock-infected cells (black)**. Differentiated HIE I124 were infected with VA1 (MOI of 1) and a single cell suspension was generated at 3 dpi. Cells were stained with the surface markers lysozyme (paneth cells), CD44 (progenitor cells), chromogranin A (enteroendocrine cells), MUC2 (goblet cells) and sucrase isomaltase (mature enterocytes) and the intracellular dsRNA antibody conjugated with biotin followed by the secondary streptavidin APC-Cy7 antibody. The flow cytometry data were generated on BD LSRFortessa and analyzed with FlowJo. The gating strategy from ‘Cells’ to ‘Single cells’ to ‘Alive cells’ is illustrated for one representative sample in the left column, whereas the gating strategy for each cell type and the corresponding infected cells is illustrated for one VA1-infected (red) and one Mock-infected (black) sample in the middle and right column, respectively.
(TIF)

S4 Fig RNAseq analysis of VA1-infected HIE. **A**) The correlation between genome copies per D124 HIE as determined by RT-qPCR and the proportion of viral transcripts in the pool of sequenced RNA collected from the same HIE cultures. **B)** IFN α, β, and λ are up-regulated upon VA1 infection in the RNAseq dataset. **C–D)** Top genes positively- **(C)** and negatively- **(D)** correlated with viral load as determined by RNA-seq and RT-qPCR.
(TIF)

S5 Fig HIE mounts IFN response to virus infection. **A)** ISG15 transcript levels at 1 dpi or post poly I:C treatment. Undifferentiated C68 HIE were infected with VA1, Sindbis virus or VSV (all MOI = 1) for 1 day or treated with 50 μg/ml poly I:C for 24 hours. Cellular RNA was extracted for ISG15 transcript quantification as fold increase over 0 dpi by qPCR. GADPH was used as internal control. **B)** VSV and Sindbis virus titer determination at 1 dpi by plaque assay with Vero cells. N ≥ 3; error = mean ± SD.
(TIF)

S1 Table RNA-seq gene summary for VA1 over mock. (DOCX)

S2 Table RNA-seq list of significantly regulated genes (Adj P < 0.05) for VA1 over mock. (DOCX)

S3 Table C_T_ values for ISG15, IFN-β, IFN-γ and IFN-λ mRNA expression in VA1 infected I124 HIE as detected by RT-qPCR. (DOCX)

S4 Table C_T_ values for ISG15, IFN-β, IFN-γ and IFN-λ mRNA expression in VA1 infected Caco2 cells as detected by RT-Qpcr. (DOCX)

S5 Table Summary of the HIE lines used in this study. (DOCX)

S6 Table List of commercial antibodies. (DOCX)

S7 Table List of primers. (DOCX)
