# Supplementary figures and images for: Astrovirus replication in human intestinal enteroids reveals multi-cellular tropism and an intricate host innate immune landscape
Source: PLoS Pathog. 2019 Oct 31;15(10):e1008057. doi: 10.1371/journal.ppat.1008057 (PMC6957189; doi:10.1371/journal.ppat.1008057)

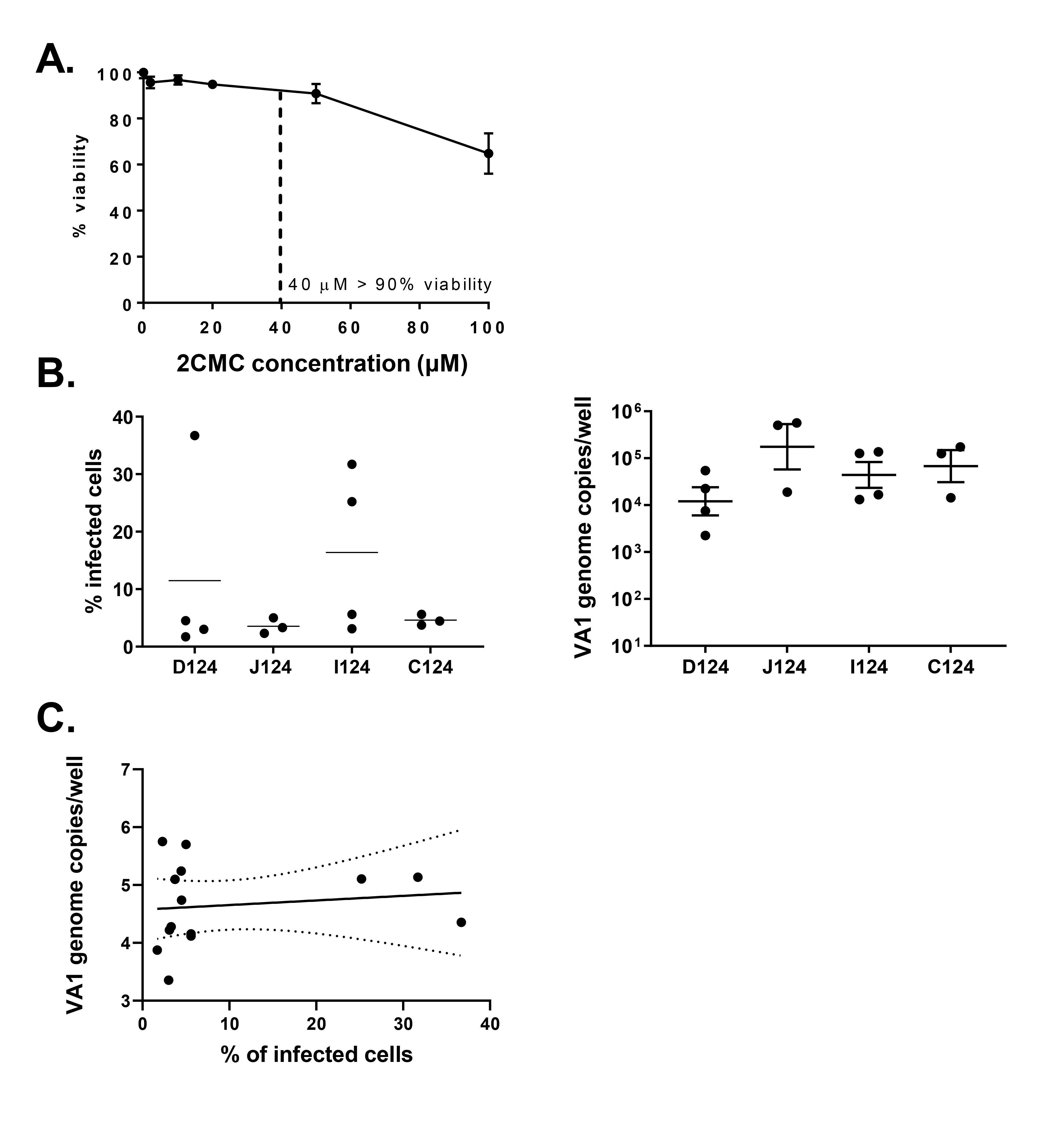

Supplement: S1 Fig — A) 2CMC is non-toxic in HIE at working concentration. Undifferentiated J2 HIE were treated with increasing concentrations of 2’-C-methylcytidine (2CMC) for 3 days (duration of VA1 infection) and cell viability was determined by WST1 assay (Roche). B) VA1 infects HIE from different compartments. A single cell suspension of VA1-infected (MOI of 1) D124, I124, J124 and C124 HIE was obtained at 3 dpi. After fixation/permeabilization, cells were stained with antibodies against dsRNA and VA1 capsid protein and analyzed by flow cytometry. Left panel represents the % of double-positive cells. On the right, the supernatants of infected HIE were harvested and RNA was extracted for RT-qPCR analysis. C) Virus genome copies and % of infected cells do not correlate. Analysis of the data represented in B and linear regression (95% confidence interval). (TIF) [file ppat.1008057.s001.tif]

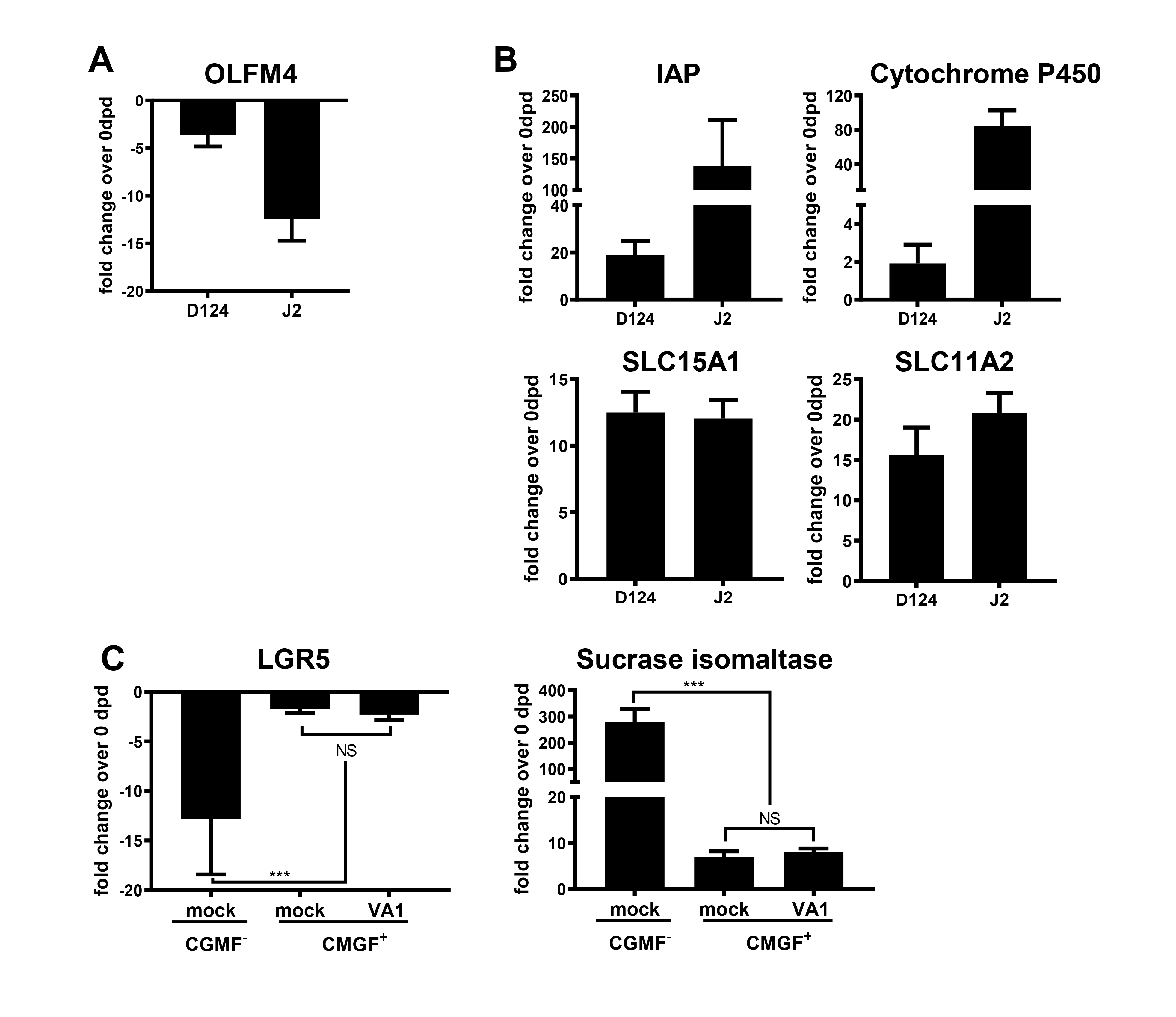

Supplement: S2 Fig — The differentiation status of J2 and D124 HIE was monitored at 0 and 6 days post-differentiation (dpd) (after WNT3A removal) by measuring transcripts of A) olfactomedin 4 (OLFM4), a stem cell marker, and B) alkaline phosphatase, cytochrome P450, SLC15A1, and SLC11A2 (markers of enterocytes). C) differentiation status of J2 HIE monitored at 0 and 3 days post differentiation after WNT3A removal (CMGF-) or VA1 infection without removal of WNT3A (CMGF+). Transcript levels were measured by qPCR. Data are from ≥ 3 experiments; error = mean ± SD. Fold change is relative to GAPDH, and is statistically significantly different (P<0.05) from the 0 dpd in A) and B). In C) ***P<0.001. Abbreviations: D = duodenum, J = jejunum. The number associated with each letter indicates the patient identifier. NS = not significant. (TIF) [file ppat.1008057.s002.tif]

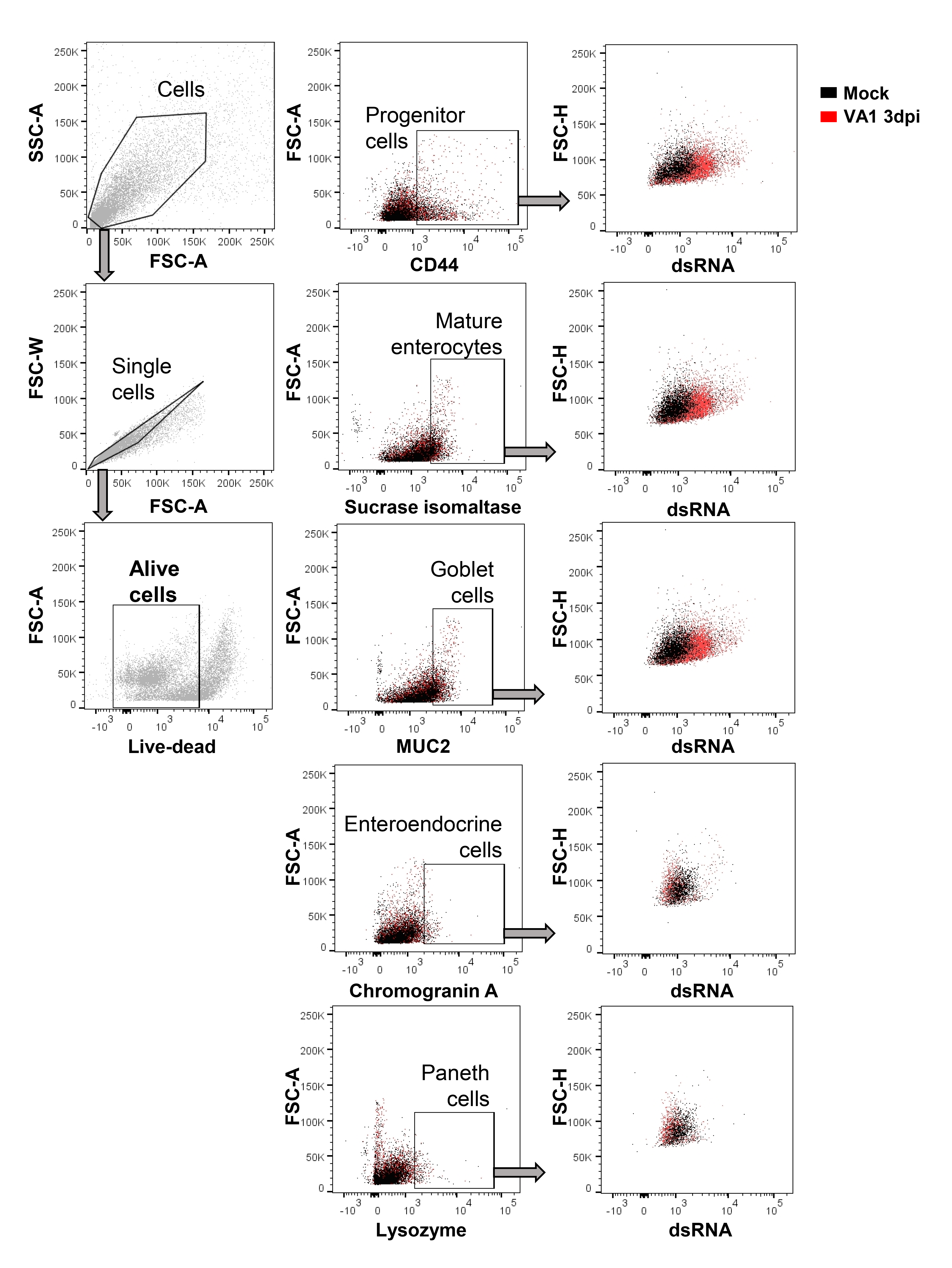

Supplement: S3 Fig — Representative flow plots of the percent of VA1-infected cells (red) vs mock-infected cells (black). Differentiated HIE I124 were infected with VA1 (MOI of 1) and a single cell suspension was generated at 3 dpi. Cells were stained with the surface markers lysozyme (paneth cells), CD44 (progenitor cells), chromogranin A (enteroendocrine cells), MUC2 (goblet cells) and sucrase isomaltase (mature enterocytes) and the intracellular dsRNA antibody conjugated with biotin followed by the secondary streptavidin APC-Cy7 antibody. The flow cytometry data were generated on BD LSRFortessa and analyzed with FlowJo. The gating strategy from ‘Cells’ to ‘Single cells’ to ‘Alive cells’ is illustrated for one representative sample in the left column, whereas the gating strategy for each cell type and the corresponding infected cells is illustrated for one VA1-infected (red) and one Mock-infected (black) sample in the middle and right column, respectively. (TIF) [file ppat.1008057.s003.tif]

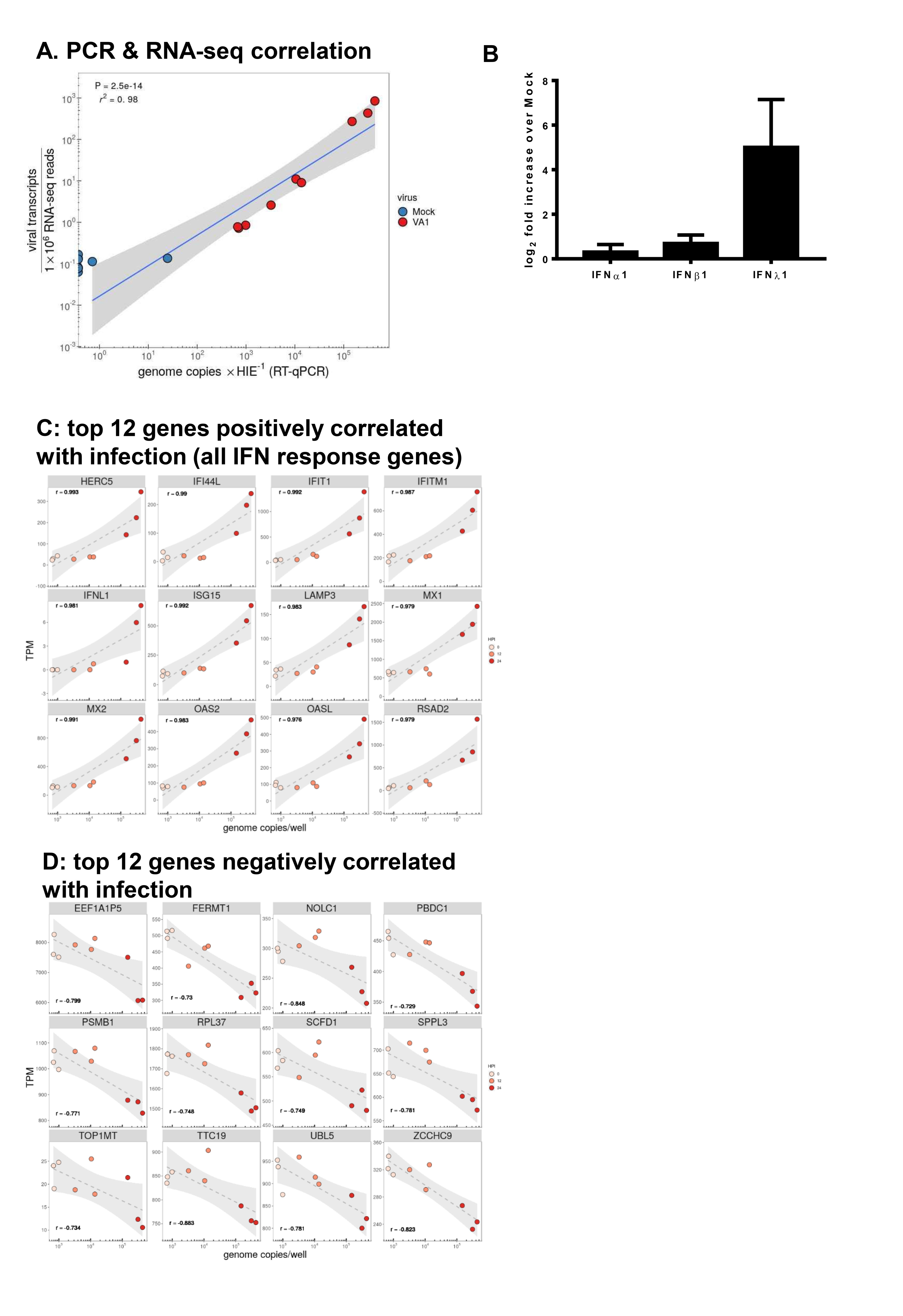

Supplement: S4 Fig — A) The correlation between genome copies per D124 HIE as determined by RT-qPCR and the proportion of viral transcripts in the pool of sequenced RNA collected from the same HIE cultures. B) IFN α, β, and λ are up-regulated upon VA1 infection in the RNAseq dataset. C–D) Top genes positively- (C) and negatively- (D) correlated with viral load as determined by RNA-seq and RT-qPCR. (TIF) [file ppat.1008057.s004.tif]

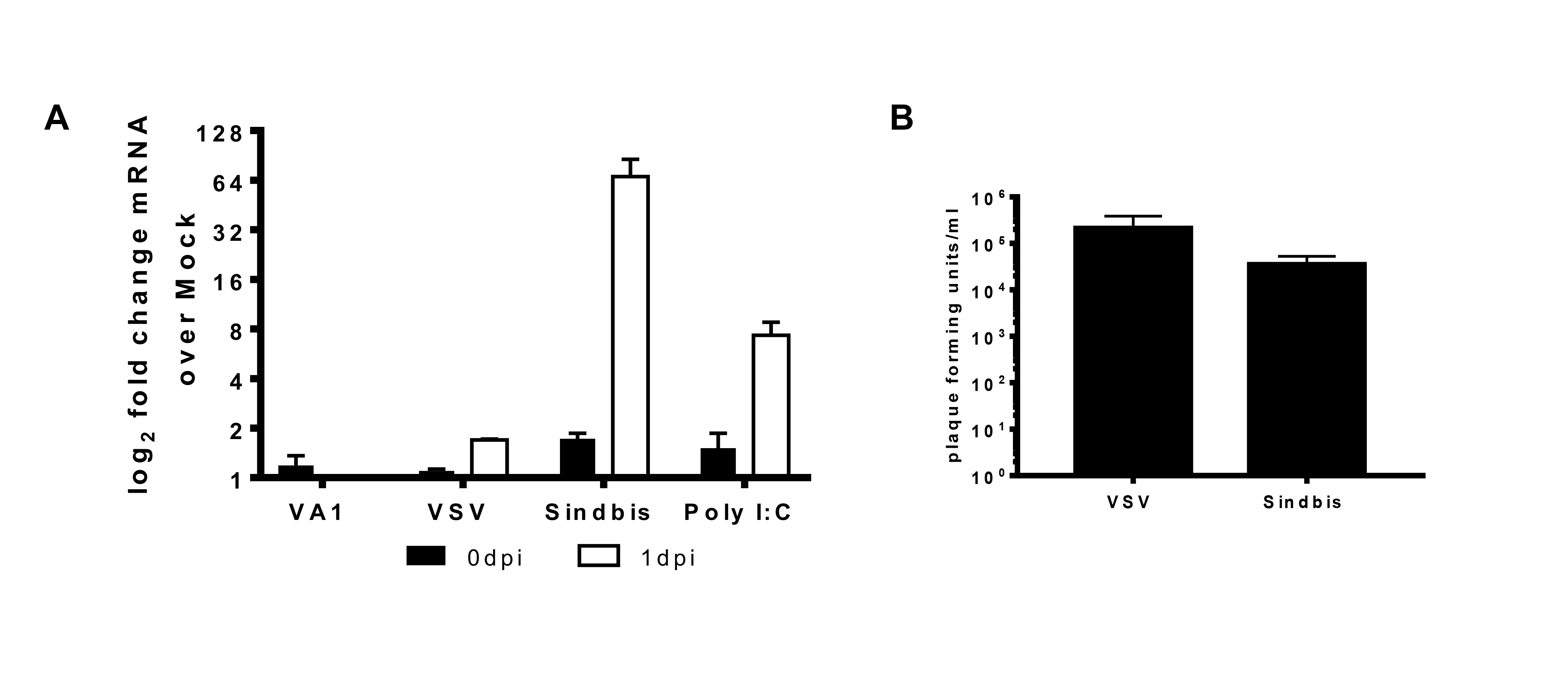

Supplement: S5 Fig — A) ISG15 transcript levels at 1 dpi or post poly I:C treatment. Undifferentiated C68 HIE were infected with VA1, Sindbis virus or VSV (all MOI = 1) for 1 day or treated with 50 μg/ml poly I:C for 24 hours. Cellular RNA was extracted for ISG15 transcript quantification as fold increase over 0 dpi by qPCR. GADPH was used as internal control. B) VSV and Sindbis virus titer determination at 1 dpi by plaque assay with Vero cells. N ≥ 3; error = mean ± SD. (TIF) [file ppat.1008057.s005.tif]
