# Supplementary material for: Astrovirus replication in human intestinal enteroids reveals multi-cellular tropism and an intricate host innate immune landscape
Source: PLoS Pathog. 2019 Oct 31;15(10):e1008057. doi: 10.1371/journal.ppat.1008057 (PMC6957189; doi:10.1371/journal.ppat.1008057)
Supplement: S1 Table — (DOCX) [file ppat.1008057.s006.docx]

**Table S1:** RNA-seq gene summary for VA1 over mock

| **hpi**^1^ | **Genes** | **Total** | **Upregulated** | **Downregulated** |
| --- | --- | --- | --- | --- |
| **0** | All genes  Adjp^2^ < 0.05 | 23220  246 | 11064  110 | 10989  136 |
| **12** | All genes  Adjp < 0.05 | 23220  13 | 11200  8 | 10798  5 |
| **24** | All genes  Adjp < 0.05 | 23220  203 | 10814  154 | 11119  49 |

^1^hpi = hours postinfection

^2^Adjp = adjusted p value
