# Supplementary material for: Astrovirus replication in human intestinal enteroids reveals multi-cellular tropism and an intricate host innate immune landscape
Source: PLoS Pathog. 2019 Oct 31;15(10):e1008057. doi: 10.1371/journal.ppat.1008057 (PMC6957189; doi:10.1371/journal.ppat.1008057)
Supplement: S2 Table — (DOCX) [file ppat.1008057.s007.docx]

**Table S2:** RNA-seq list of significantly regulated genes (Adj P < 0.05) for VA1 over mock

| **Upregulated** | | |  | **Downregulated** | | |
| --- | --- | --- | --- | --- | --- | --- |
| **0 hpi** | **12 hpi** | **24 hpi** |  | **0 hpi** | **12 hpi** | **24 hpi** |
| AC106886.5 | CPSF6 | ACHE |  | ADNP2 | ANKRD36 | ACTR2 |
| AC132008.2 | DDR1 | ADAR |  | AGR2 | H1F0 | AKAP10 |
| ACHE | KMT2D | AGAP4 |  | AGR3 | LARS | ANXA1 |
| AMY2B | MGAT4B | AL365205.1 |  | AHNAK | PLCG2 | ARL1 |
| ANPEP | MT-ND1 | ALS2CL |  | ANXA1 | SYNPR | BZW1 |
| ATG4B | MT-ND2 | ANKZF1 |  | ANXA10 |  | CBWD1 |
| BMF | MTND2P28 | ANO9 |  | ANXA13 |  | CD59 |
| C15orf52 | SLC37A1 | AP1G2 |  | ARCN1 |  | CDV3 |
| C19orf66 |  | ATG16L2 |  | ARF4 |  | CEACAM6 |
| CA13 |  | ATG4B |  | ARFGEF3 |  | COPB1 |
| CASZ1 |  | ATN1 |  | ARHGDIB |  | CREB1 |
| CBLB |  | BATF2 |  | ATP10D |  | DNAJC10 |
| CCDC183 |  | BAZ2A |  | ATP5A1 |  | DNTTIP2 |
| CDHR2 |  | BICDL2 |  | ATP5B |  | EFNB2 |
| CDHR5 |  | BOP1 |  | ATP5G3 |  | EIF4G2 |
| CDKN1B |  | BRD4 |  | ATP5H |  | FERMT1 |
| CNOT6L |  | BST2 |  | ATP7B |  | FP565260.7 |
| CREB3L1 |  | C19orf66 |  | BCAS1 |  | G3BP1 |
| CYP2C9 |  | CAPN8 |  | C1orf116 |  | GLOD4 |
| CYP4F12 |  | CCDC183 |  | CA2 |  | GPD2 |
| DAB2 |  | CCDC57 |  | CALU |  | GSK3B |
| DGKA |  | CDK18 |  | CCT4 |  | HNRNPA3 |
| DGKZ |  | CHKB-CPT1B |  | CKAP5 |  | ITGA2 |
| DMAP1 |  | CMPK2 |  | CLIC1 |  | ITGB1 |
| ECHDC2 |  | CMTR1 |  | COX6A1 |  | KIAA0232 |
| EFNA1 |  | DCAF5 |  | COX7C |  | MAPRE1 |
| EPS8L2 |  | DDX58 |  | CYB561 |  | MORF4L1 |
| ERBB3 |  | DDX60 |  | CYB5B |  | MT-ND3 |
| FAM111A |  | DDX60L |  | CYBA |  | MTF1 |
| FBRS |  | DGKA |  | DDB1 |  | MTMR1 |
| FLCN |  | DHX58 |  | DHCR24 |  | MYO1E |
| FTH1 |  | ECHDC2 |  | DLAT |  | N4BP2 |
| GABRE |  | EIF2AK2 |  | DNAJB1 |  | NQO1 |
| GOLGA6L9 |  | EPN1 |  | DUSP4 |  | PLS1 |
| GOLGA8A |  | EPOR |  | DUSP5 |  | RAB3IP |
| GRB7 |  | EPS8L2 |  | EEF1A1 |  | RASSF6 |
| GSDMB |  | EPSTI1 |  | EEF1G |  | RPS27L |
| HNRNPH1 |  | ETV7 |  | EFCAB14 |  | RPS6KA3 |
| HOOK2 |  | FER1L4 |  | EIF4A1 |  | SEC63 |
| ICA1 |  | FLCN |  | EIF4G2 |  | SEPT7 |
| ID3 |  | GABBR1 |  | EIF4H |  | SLC7A11 |
| IL32 |  | GABRE |  | EZR |  | SLX1B |
| JMY |  | GBP1 |  | FOSL2 |  | TMEM87B |
| KIAA1217 |  | GOLGA8A |  | GCNT3 |  | TPM4 |
| LETM1 |  | GRINA |  | H2AFZ |  | TXNRD1 |
| LGALS9C |  | HELZ2 |  | H3F3B |  | UBE2H |
| LINC01347 |  | HERC5 |  | HAS3 |  | UGDH |
| LRP4 |  | HERC6 |  | HMGCR |  | ZBED9 |
| MAF |  | HOOK2 |  | HSPA8 |  | ZCCHC9 |
| MAP2K2 |  | HSH2D |  | HSPD1 |  |  |
| MBD6 |  | IFI16 |  | ISY1-RAB43 |  |  |
| MED15 |  | IFI27 |  | ITGA2 |  |  |
| MICAL3 |  | IFI35 |  | KIF5B |  |  |
| MTMR11 |  | IFI44 |  | KLF13 |  |  |
| MUC20 |  | IFI44L |  | KRT19 |  |  |
| MUC20P1 |  | IFI6 |  | KRT19P1 |  |  |
| MYO15B |  | IFIH1 |  | KRT8P36 |  |  |
| NEDD4 |  | IFIT1 |  | KRTCAP3 |  |  |
| NFKBIZ |  | IFIT2 |  | LDHB |  |  |
| NIPBL |  | IFIT3 |  | LDLR |  |  |
| NOL4L |  | IFITM1 |  | LGALS4 |  |  |
| NPEPPS |  | IFITM3 |  | LRBA |  |  |
| NPIPB14P |  | IRF7 |  | MAFF |  |  |
| OSBPL7 |  | ISG15 |  | MAPRE1 |  |  |
| PARP6 |  | JMJD7-PLA2G4B |  | MGAT3 |  |  |
| PCGF2 |  | KAT2B |  | MT-CO1 |  |  |
| PCK2 |  | KCNC3 |  | MT-CO2 |  |  |
| PDXDC2P |  | KIAA1522 |  | MT-CO3 |  |  |
| PHYKPL |  | KIF12 |  | MTATP6P1 |  |  |
| PI4KAP2 |  | KIFC2 |  | MYCBP2 |  |  |
| PIGZ |  | LAMB2 |  | MYNN |  |  |
| PLA2G6 |  | LAMP3 |  | NDUFA4 |  |  |
| PLEKHA7 |  | LAP3 |  | NOMO3 |  |  |
| POU5F1 |  | LENG8 |  | NQO1 |  |  |
| RAB11FIP3 |  | LGALS9 |  | NSDHL |  |  |
| RBM6 |  | LINC01347 |  | NT5C |  |  |
| REC8 |  | LPIN3 |  | PANK3 |  |  |
| RERE |  | LRCH4 |  | PCSK9 |  |  |
| RNF44 |  | LRP4 |  | PGAM1 |  |  |
| SCAMP4 |  | MBD6 |  | PPIA |  |  |
| SDHAP1 |  | MBOAT7 |  | PRKCI |  |  |
| SERINC2 |  | MLPH |  | PSMB2 |  |  |
| SF1 |  | MROH1 |  | PTGR1 |  |  |
| SH2B1 |  | MSH5 |  | RAB25 |  |  |
| SLC17A4 |  | MST1 |  | REEP5 |  |  |
| SLC25A29 |  | MTMR11 |  | REPS2 |  |  |
| SLC25A37 |  | MX1 |  | RHPN2 |  |  |
| SLC26A6 |  | MX2 |  | RPL10A |  |  |
| SMAD7 |  | MYO15B |  | RPL12 |  |  |
| SPPL2B |  | MYO1A |  | RPL13A |  |  |
| SRRM2 |  | NPAS2 |  | RPL14 |  |  |
| STARD7 |  | OAS1 |  | RPL15 |  |  |
| STK36 |  | OAS2 |  | RPL19 |  |  |
| TEPSIN |  | OAS3 |  | RPL21P16 |  |  |
| TIMP2 |  | OASL |  | RPL23A |  |  |
| TMEM185A |  | OGFR |  | RPL27 |  |  |
| TMEM198B |  | OPTN |  | RPL27A |  |  |
| TRIM31 |  | PAQR5 |  | RPL29 |  |  |
| TTLL3 |  | PARP10 |  | RPL3 |  |  |
| TYK2 |  | PARP12 |  | RPL32 |  |  |
| UVSSA |  | PARP9 |  | RPL4 |  |  |
| VEGFA |  | PDZD3 |  | RPL5 |  |  |
| VIPR1 |  | PHYKPL |  | RPL7 |  |  |
| WSB1 |  | PI4KAP2 |  | RPL7A |  |  |
| ZFP36L1 |  | PLEKHG2 |  | RPL9P9 |  |  |
| ZNF12 |  | PLEKHH3 |  | RPLP0 |  |  |
| ZNF224 |  | PML |  | RPS12 |  |  |
| ZNF3 |  | PNPLA6 |  | RPS18 |  |  |
| ZNF488 |  | PNPT1 |  | RPS24 |  |  |
| ZZEF1 |  | PTPN14 |  | RPS3A |  |  |
|  |  | RAB17 |  | RPS4X |  |  |
|  |  | RBM6 |  | RPSA |  |  |
|  |  | RDH13 |  | S100A6 |  |  |
|  |  | REC8 |  | SERPINB8 |  |  |
|  |  | RNF19A |  | SLC16A1 |  |  |
|  |  | RNF207 |  | SLC35B2 |  |  |
|  |  | RNF213 |  | SLC9A7 |  |  |
|  |  | RNF31 |  | SPINK1 |  |  |
|  |  | RSAD2 |  | SPTLC2 |  |  |
|  |  | RTP4 |  | ST6GALNAC1 |  |  |
|  |  | RUBCN |  | STARD10 |  |  |
|  |  | SAMD9 |  | SYTL5 |  |  |
|  |  | SAMHD1 |  | TAGLN2 |  |  |
|  |  | SCAMP4 |  | TFRC |  |  |
|  |  | SLC22A18 |  | TGFBR2 |  |  |
|  |  | SLC25A28 |  | TGS1 |  |  |
|  |  | SRRM2 |  | TMED9 |  |  |
|  |  | STAT1 |  | TMTC2 |  |  |
|  |  | STAT2 |  | TMX2 |  |  |
|  |  | STK11IP |  | TPM3 |  |  |
|  |  | STOX2 |  | TSPAN5 |  |  |
|  |  | SUN2 |  | TXNIP |  |  |
|  |  | SYT8 |  | UQCRC2 |  |  |
|  |  | TAF1C |  | VIL1 |  |  |
|  |  | TAP1 |  | XBP1 |  |  |
|  |  | TMEM198B |  | XRCC6 |  |  |
|  |  | TMEM94 |  |  |  |  |
|  |  | TOR1B |  |  |  |  |
|  |  | TREX1 |  |  |  |  |
|  |  | TRIM21 |  |  |  |  |
|  |  | TRIM25 |  |  |  |  |
|  |  | TTLL3 |  |  |  |  |
|  |  | UBA7 |  |  |  |  |
|  |  | UBE2L6 |  |  |  |  |
|  |  | UCKL1 |  |  |  |  |
|  |  | UNC93B1 |  |  |  |  |
|  |  | USP18 |  |  |  |  |
|  |  | VEGFA |  |  |  |  |
|  |  | WSB1 |  |  |  |  |
|  |  | XAF1 |  |  |  |  |
|  |  | YEATS2 |  |  |  |  |
|  |  | ZBP1 |  |  |  |  |
|  |  | ZC3HAV1 |  |  |  |  |
|  |  | ZNF76 |  |  |  |  |
