# Supplementary material for: Astrovirus replication in human intestinal enteroids reveals multi-cellular tropism and an intricate host innate immune landscape
Source: PLoS Pathog. 2019 Oct 31;15(10):e1008057. doi: 10.1371/journal.ppat.1008057 (PMC6957189; doi:10.1371/journal.ppat.1008057)
Supplement: S3 Table — (DOCX) [file ppat.1008057.s008.docx]

**Table S3**: C_T_ values for ISG15, IFN-β, IFN-γ and IFN-λ mRNA expression in VA1 infected I124 HIE as detected by RT-qPCR

| **I124** |  |  |  |  |  |  |
| --- | --- | --- | --- | --- | --- | --- |
| **dpi** | **virus** | **GAPDH** | **ISG15** | **IFN-β** | **IFN-γ** | **IFN-λ** |
| **0** | **mock** | 16.45894 | 25.17327 | 30.04527 | 34.57608 | 34.94001 |
|  | **VA1** | 17.07058 | 25.70306 | 31.3828 | 36.50319 | 36.15627 |
| **1** | **mock** | 16.85144 | 25.19652 | 31.91958 | 35.97657 | 35.78828 |
|  | **VA1** | 17.18724 | 21.20768 | 26.26553 | 34.61571 | 27.17043 |
| **2** | **mock** | 16.30459 | 25.98087 | 31.66673 | 35.56068 | 36.20036 |
|  | **VA1** | 17.12754 | 17.16788 | 25.64154 | 34.27983 | 26.85919 |
| **3** | **mock** | 16.83636 | 25.76905 | 29.36646 | 33.88408 | 34.18761 |
|  | **VA1** | 16.87216 | 18.77343 | 25.01613 | 32.98357 | 24.52153 |
|  |  |  |  |  |  |  |
|  |  | **GAPDH** | **ISG15** | **IFN-β** | **IFN-γ** | **IFN-λ** |
| **0** | **mock** | 16.3934 | 25.579 | 32.06571 | 34.91685 | 34.01713 |
|  | **VA1** | 16.90018 | 26.49462 | 31.03896 | 34.177 | 34.79215 |
| **1** | **mock** | 16.80226 | 26.87604 | 32.99203 | 34.42669 | 33.54849 |
|  | **VA1** | 16.53441 | 20.07964 | 26.73478 | 33.92269 | 29.12171 |
| **2** | **mock** | 16.94168 | 25.45018 | 31.18915 | 33.93393 | 33.06908 |
|  | **VA1** | 16.6129 | 18.93752 | 27.16829 | 35.29465 | 26.65605 |
| **3** | **mock** | 16.25046 | 24.79665 | 33.59433 | 35.73599 | 34.45672 |
|  | **VA1** | 16.43137 | 17.83876 | 26.25061 | 33.26504 | 26.36023 |

dpi = days post infection
