# Supplementary material for: Astrovirus replication in human intestinal enteroids reveals multi-cellular tropism and an intricate host innate immune landscape
Source: PLoS Pathog. 2019 Oct 31;15(10):e1008057. doi: 10.1371/journal.ppat.1008057 (PMC6957189; doi:10.1371/journal.ppat.1008057)
Supplement: S4 Table — (DOCX) [file ppat.1008057.s009.docx]

**Table S4**: C_T_ values for ISG15, IFN-β, IFN-γ and IFN-λ mRNA expression in VA1 infected Caco2 cells as detected by RT-qPCR

| **Caco2** |  |  |  |  |  |  |
| --- | --- | --- | --- | --- | --- | --- |
| **dpi** | **virus** | **GAPDH** | **ISG15** | **IFN-β** | **IFN-γ** | **IFN-λ** |
| **0** | **mock** | 16.52736 | 24.49094 | 29.68363 | 32.06176 | 30.26431 |
|  | **VA1** | 17.29411 | 24.72471 | 28.63101 | 31.7208 | 29.12303 |
| **1** | **mock** | 16.36817 | 24.15891 | 28.67521 | 31.93361 | 29.79098 |
|  | **VA1** | 17.31171 | 25.14748 | 29.47564 | 32.20574 | 31.03646 |
| **2** | **mock** | 16.71192 | 24.16807 | 29.15547 | 32.26011 | 30.16558 |
|  | **VA1** | 16.33313 | 24.06912 | 29.11841 | 32.07088 | 28.93904 |
| **3** | **mock** | 16.41145 | 23.59377 | 28.8531 | 32.22242 | 29.40009 |
|  | **VA1** | 16.4882 | 23.83544 | 29.84037 | 32.20444 | 30.50532 |
|  |  |  |  |  |  |  |
|  |  | **GAPDH** | **ISG15** | **IFN-β** | **IFN-γ** | **IFN-λ** |
| **0** | **mock** | 16.82736 | 24.89094 | 30.68363 | 33.06176 | 33.26431 |
|  | **VA1** | 16.40293 | 24.58243 | 30.24919 | 34.34448 | 34.36169 |
| **1** | **mock** | 16.20226 | 24.44114 | 32.12706 | 34.15401 | 32.91231 |
|  | **VA1** | 16.10231 | 23.73528 | 30.27891 | 33.94943 | 32.33202 |
| **2** | **mock** | 15.73636 | 23.31885 | 28.95103 | 33.86556 | 30.89455 |
|  | **VA1** | 16.12894 | 23.53304 | 28.57402 | 32.90056 | 29.82342 |
| **3** | **mock** | 15.95577 | 23.29778 | 29.5803 | 33.78662 | 30.72195 |
|  | **VA1** | 16.08243 | 23.23364 | 28.29216 | 32.10407 | 29.67912 |

dpi = days post infection
