# Supplementary material for: Astrovirus replication in human intestinal enteroids reveals multi-cellular tropism and an intricate host innate immune landscape
Source: PLoS Pathog. 2019 Oct 31;15(10):e1008057. doi: 10.1371/journal.ppat.1008057 (PMC6957189; doi:10.1371/journal.ppat.1008057)
Supplement: S5 Table — (DOCX) [file ppat.1008057.s010.docx]

**Table S5:** Summary of the HIE lines used in this study

| **ID** | ***Intestinal segment*** | ***Patient ID*** | ***Age*** | ***Sex*** | ***Medical condition*** |
| --- | --- | --- | --- | --- | --- |
| **D124** | Duodenum | 124 | 116 days | M | Normal |
| **J124** | Jejunum | 124 | 116 days | M | Normal |
| **I124** | Ileum | 124 | 116 days | M | Normal |
| **C124** | Colon | 124 | 116 days | M | Normal |
| **C68** | Colon | 68 | 87 days | M | Normal |
| **C143** | Colon | 143 |  |  |  |
|  |  |  |  |  |  |
| **D115** | Duodenum | 115 | 46 yo^1^ | F | Normal |
| **D87** | Duodenum | 87 | 21 yo | M | Normal |
| **C87** | Colon | 87 | 21 yo | M | Normal |
| **J2** | Jejunum | 2 | NA^2^ | NA | NA |
| **I104** | Ileum | 104 | 51 yo | M | Normal |
| **C89** | Colon | 89 | 55 yo | M | Normal |

^1^yo = years old; ^2^NA = information not available
