# Supplementary material for: Astrovirus replication in human intestinal enteroids reveals multi-cellular tropism and an intricate host innate immune landscape
Source: PLoS Pathog. 2019 Oct 31;15(10):e1008057. doi: 10.1371/journal.ppat.1008057 (PMC6957189; doi:10.1371/journal.ppat.1008057)
Supplement: S6 Table — (DOCX) [file ppat.1008057.s011.docx]

**Table S6**: List of commercial antibodies

|  | **Antibody name** | **Company** | **Cat. No.** | **Dilution** |
| --- | --- | --- | --- | --- |
| **Flow cytometry** | BV421 mouse anti-human CD44 | BD bioscience | 562890 | 1:200 |
|  | Mucin-2 (F-2) FITC | Santa Crux biotechnology | 515032 | 1:400 |
|  | Sucrase-Isomaltase (A-12) PE | Santa Crux biotechnology | 393424 | 1:100 |
|  | APC-human lysozyme Ab | AssayPro | 30331-05161 | 1:100 |
|  | Chromogranin A Antibody PerCP | Novus Biologicals | 33198PCP | 1:50 |
|  | anti-mouse VP1-VA1 | Janowski lab (Washington University) | - | 1:100 |
|  | dsRNA-biotinilated | Scicons | J2 | 1:50 |
|  | Streptavidin APC-Cy™7 | eBioscience | 47431782 | 1:2000 |
| **Compensation** | BV421 anti-human CD45 (HI30) | BioLegend | 304031 | 1:200 |
|  | Anti-human CD45 FITC (HI30) | BioLegend | 555482 | 1:400 |
|  | PE anti-human CD45 (2D1) | BD bioscience | 368509 | 1:100 |
|  | APC anti-human CD45 (2D1) | BD bioscience | 368511 | 1:100 |
|  | PerCP anti-human CD19 (HIB19) | BioLegend | 302227 | 1:50 |
| **Immunofluorescence** | Fluorescein labeled *Ulex Europaeus* Agglutinin I (UEA I) | Vector Laboratories | FL - 1061-5 | 1:1000 |
|  | ZO-1 Rabbit Polyclonal Antibody | Invitrogen | 617300 | 1:500 |
|  | VA1 hyperimmune polyclonal serum | Wobus Lab | - | 1:500 |
|  | dsRNA | Scicons | J2 | 1:400 |
|  | Intestinal-type alkaline phosphatase antibody, rabbit anti-human (IAP) | Biorbyt | orb241671 | 1:400 |
|  | OLMF4 rabbit polyclonal antibody | Abcam | ab85046 | 1:400 |
|  | AlexaFluor 594 goat anti-mouse | Life technology |  | 1:1000 |
|  | AlexaFluor 647 goat anti-rabbit | Life technology |  | 1:1000 |
|  |  |  |  |  |
| **Western blotting** | Rabbit anti ISG15 | Cell Signaling | 2743S | 1:1000 |
|  | Mouse anti β-actin | Cell Signaling | 3700S | 1:10000 |
|  | IRDye 800CW Goat-anti mouse | LI-COR | 926-32210 | 1:10000 |
|  | IRDye 680RD Goat-anti-rabbit | LI-COR | 926-68071 | 1:10000 |
