# Supplementary material for: Astrovirus replication in human intestinal enteroids reveals multi-cellular tropism and an intricate host innate immune landscape
Source: PLoS Pathog. 2019 Oct 31;15(10):e1008057. doi: 10.1371/journal.ppat.1008057 (PMC6957189; doi:10.1371/journal.ppat.1008057)
Supplement: S7 Table — (DOCX) [file ppat.1008057.s012.docx]

**Table S7**: List of primers

| **Name** | **Sequence 5’ – 3’** | | **Ref** |
| --- | --- | --- | --- |
|  | **Forward** | **Reverse** |  |
| HAstV | CCAGRCTCACAGAAGAGCAAC | CTTGCTAGCCATCRCACTTCTT | ^1^ |
|  | Probe: 6-FAM-CATCGCATTTGGAGGGGAGGACC-BHQ1 | |  |
| MLB1 | TGCGGTGTCGCAGACAAT | AAAGACTGCACATTCAGTTTCAACA | ^1^ |
|  | Probe: 6-FAM-CAGCAACACTCGGCACCGTTGG-BHQ1 | |  |
| VA1 | CTAGTGGTGGGGAAGAAC | CCTTGGCTATTTGCTTTGC | ^2^ |
|  | Probe: 56-FAM/CCATGACTT/ZEN/TGCTTTGGACCTCCC/3IABkFQ | |  |
| ALPI | CATACCTGGCTCTGTCCAAGA | GTCTGGAAGTTGGCCTTGAC | ^3^ |
| CYP3A4 | GATGGCTCTCATCCCAGACTT | AGTCCATGTGAATGGGTTCC | ^3^ |
| SLC15A1 | TCTCTGTCACGGGATTGGA | CTGCCTGAAGCACCGACT | ^3^ |
| SLC11A2 | CACCGTCAGTATCCCAAGGT | CCGATGATAGCCAACTCCAC | ^3^ |
| SI | AATCCTTTTGGCATCCAGATT | GCAGCCAAGAATCCCAAAT | ^3^ |
| MUC2 | TGTAGGCATCGCTCTTCTCA | GACACCATCTACCTCACCCG | ^4^ |
| LYSOZYME | ACAAGCTACAGCATCAGCGA | GTAATGATGGCAAAACCCCA | ^5^ |
| OLFM4 | ACCTTTCCCGTGGACAGAGT | TGGACATATTCCCTCACTTTGGA | ^5^ |
| LGR5 | CAGCGTCTTCACCTCCTACC | TGGGAATGTATGTCAGAGCG | ^6^ |
| GAPDH | CTCTGCTCCTCCTGTTCGAC | TTAAAAGCAGCCCTGGTGAC | ^4^ |
| IFN-β | TTGACATCCCTGAGGAGATTAAGC | TTAGCCAGGAGGTTCTCAACAATAG | ^7^ |
| IFN-γ | TTGGAAAGAGGAGAGTGACAG | ACATTCATGTCTTCCTTGATGG | ^7^ |
| IFN-λ | GTTCAAATCTCTGTCACCAC | TTCAGCTTGAGTGACTCTTC | ^7^ |
| ISG15 | TGGCGGGCAACGAATT | GGGTGATCTGCGCCTTCA | ^8^ |
| MX1 | CCAGCTGCTGCATCCCACCC | AGGGGCGCACCTTCTCCTCA | ^9^ |
| OAS2 | ACCCGAACAGTTCCCCCTGGT | ACAAGGGTACCATCGGAGTTGCC | ^10^ |
| Viperin | TGCCACAATGTGGGTGCTTACAC | CTCAAGGGGCAGCACAAAGGAT | ^11^ |

**References for Table S7:**

1 Gu, Z. *et al.* Comparative Evaluation of Broad-Panel PCR Assays for the Detection of Gastrointestinal Pathogens in Pediatric Oncology Patients. *J Mol Diagn* **17**, 715-721, doi:10.1016/j.jmoldx.2015.06.003 (2015).

2 Janowski, A. B., Bauer, I. K., Holtz, L. R. & Wang, D. Propagation of astrovirus VA1, a neurotropic human astrovirus, in cell culture. *J Virol*, doi:10.1128/JVI.00740-17 (2017).

3 Natoli, M., Leoni, B. D., D'Agnano, I., Zucco, F. & Felsani, A. Good Caco-2 cell culture practices. *Toxicol In Vitro* **26**, 1243-1246, doi:10.1016/j.tiv.2012.03.009 (2012).

4 Tsai, Y. H. *et al.* In vitro patterning of pluripotent stem cell-derived intestine recapitulates in vivo human development. *Development* **144**, 1045-1055, doi:10.1242/dev.138453 (2017).

5 Finkbeiner, S. R. *et al.* Transcriptome-wide Analysis Reveals Hallmarks of Human Intestine Development and Maturation In Vitro and In Vivo. *Stem Cell Reports*, doi:10.1016/j.stemcr.2015.04.010 (2015).

6 Tsai, Y. H. *et al.* LGR4 and LGR5 Function Redundantly During Human Endoderm Differentiation. *Cell Mol Gastroenterol Hepatol* **2**, 648-662 e648, doi:10.1016/j.jcmgh.2016.06.002 (2016).

7 Hillyer, P. *et al.* Expression profiles of human interferon-alpha and interferon-lambda subtypes are ligand- and cell-dependent. *Immunol Cell Biol* **90**, 774-783, doi:10.1038/icb.2011.109 (2012).

8 Scagnolari, C. *et al.* ISG15 expression correlates with HIV-1 viral load and with factors regulating T cell response. *Immunobiology* **221**, 282-290, doi:10.1016/j.imbio.2015.10.007 (2016).

9 Pautasso, S. *et al.* Strategy of Human Cytomegalovirus To Escape Interferon Beta-Induced APOBEC3G Editing Activity. *J Virol* **92**, doi:10.1128/JVI.01224-18 (2018).

10 Nogimori, T. *et al.* Dom34 mediates targeting of exogenous RNA in the antiviral OAS/RNase L pathway. *Nucleic Acids Res* **47**, 432-449, doi:10.1093/nar/gky1087 (2019).

11 Diget, E. A. *et al.* Characterization of HIV-1 infection and innate sensing in different types of primary human monocyte-derived macrophages. *Mediators Inflamm* **2013**, 208412, doi:10.1155/2013/208412 (2013).
